# Genome Organization of Four Brazilian Xanthomonas albilineans Strains Does Not Correlate with Aggressiveness

**DOI:** 10.1128/spectrum.02802-22

**Published:** 2023-04-13

**Authors:** Raquel P. Miranda, Paula C. G. Turrini, Dora T. Bonadio, Marcelo M. Zerillo, Arthur P. Berselli, Silvana Creste, Marie-Anne Van Sluys

**Affiliations:** a Departamento de Botânica, Instituto de Biociências, Universidade de São Paulo (USP), Butanta, São Paulo, Brazil; b Centro de Cana, Instituto Agronômico de Campinas (IAC), Campinas, São Paulo, Brazil; USDA - San Joaquin Valley Agricultural Sciences Center

**Keywords:** comparative genomics, *in vitro* transcriptome, mobile genetic elements, SPI-1 T3SS, pangenome, whole-genome SNP, Brazilian isolates, sugarcane fields

## Abstract

An integrative approach combining genomics, transcriptomics, and cell biology is presented to address leaf scald disease, a major problem for the sugarcane industry. To gain insight into the biology of the causal agent, the complete genome sequences of four Brazilian Xanthomonas albilineans strains with differing virulence capabilities are presented and compared to the GPEPC73 reference strain and FJ1. Based on the aggressiveness index, different strains were compared: Xa04 and Xa11 are highly aggressive, Xa26 is intermediate, and Xa21 is the least, while, based on genome structure, Xa04 shares most of its genomic features with Xa26, and Xa11 share most of its genomic features with Xa21. In addition to presenting more clustered regularly interspaced short palindromic repeats (CRISPR) clusters, four more novel prophage insertions are present than the previously sequenced GPEPC73 and FJ1 strains. Incorporating the aggressiveness index and *in vitro* cell biology into these genome features indicates that disease establishment is not a result of a single determinant factor, as in most other *Xanthomonas* species. The Brazilian strains lack the previously described plasmids but present more prophage regions. In pairs, the most virulent and the least virulent share unique prophages. *In vitro* transcriptomics shed light on the 54 most highly expressed genes among the 4 strains compared to ribosomal proteins (RPs), of these, 3 outer membrane proteins. Finally, comparative albicidin inhibition rings and *in vitro* growth curves of the four strains also do not correlate with pathogenicity. In conclusion, the results disclose that leaf scald disease is not associated with a single shared characteristic between the most or the least pathogenic strains.

**IMPORTANCE** An integrative approach is presented which combines genomics, transcriptomics, and cell biology to address leaf scald disease. The results presented here disclose that the disease is not associated with a single shared characteristic between the most pathogenic strains or a unique genomic pattern. Sequence data from four Brazilian strains are presented that differ in pathogenicity index: Xa04 and Xa11 are highly virulent, Xa26 is intermediate, and Xa21 is the least pathogenic strain, while, based on genome structure, Xa04 shares with Xa26, and Xa11 shares with X21 most of the genome features. Other than presenting more CRISPR clusters and prophages than the previously sequenced strains, the integration of aggressiveness and cell biology points out that disease establishment is not a result of a single determinant factor as in other xanthomonads.

## INTRODUCTION

*Xanthomonas* is a Gram-negative *Proteobacteria* lineage with several phytopathogenic strains responsible for huge losses in grain production, including rice as well as vegetable crops (1–3). These bacteria are distributed worldwide and invade their hosts through natural apertures such as stomata and hydathodes, by entering through wounds on plant tissues, or by hitchhiking on sap-feeding insects and others (2, 4). Once the bacteria escape the host immune system, they colonize plant intercellular spaces or are dispersed systemically by moving through the transport vessels in the plants (2). Among the species, Xanthomonas albilineans causes leaf scald, a systemic disease that affects plants of the *Poaceae* family, especially sugarcane (*Saccharum* spp.), resulting in substantial losses in the sugarcane field productivity (4–6). Infected plants can manifest the disease symptoms at three different stages, including latent, characterized by the absence of external symptoms; chronic, characterized by the presence of multiple symptoms, such as the appearance of white streaks, lateral leaf blade chlorosis, necrosis, and death of the shoots; and acute infection, characterized by the leaf scald, from which the name of the disease is derived (5, 7, 8), that culminates with plant death. Moreover, there is no effective treatment for the disease, so prophylaxis and crop management are the best strategies to circumvent this condition (7). To date, the most effective method to recover an infected genotype is by meristem microtip culture, followed by indexation of the plants by sensitive diagnostic methods. Therefore, the use of disease-free seed cane in sugarcane nurseries followed by crop management in the field is the best practice to restrain disease propagation (7; S. Creste, personal communication).

X. albilineans is unique in the *Xanthomonas* genus. It is recognized for its broad host range and the absence of the classical host-pathogen interaction whereby resistance gene introgression could naturally be developed, including the lack of pathogenic and adaptation gene clusters that are common in the genomes of the other *Xanthomonadaceae* (9). The X. albilineans sugarcane pathosystem is not the result of the expression of typical effector molecules produced by the bacteria that interact with resistant, or susceptible, genes in the plant but the interaction between the responses driven by the complex hybrid sugarcane genome and bacterial strains that differ in virulence but share similar genomes (3, 10). Comparative studies among isolates from Guadeloupe Island (11) and other world geographical regions (12) have shown that, despite the high genomic similarity, the observed wide range of virulence between its strains confirms the existence of different pathotypes coexisting in the same geographical region. Recent genomic studies compared X. albilineans JG43 and Xanthomonas sacchari DD13 suggest quorum sensing is an important mediator of cellular communication (10). In addition, previous studies carried out by the Centro da Cana (Campinas Agronomic Institute [IAC]) detected polymorphisms when short sequence repeat markers were screened across 44 Brazilian isolates of X. albilineans, with reported different levels of aggressiveness when inoculated in the same susceptible sugarcane host (8). No marker was correlated with the phenotypes, with the exception of the association of two simple sequence repeat (SSR) fragments with some of the most virulent isolates. Therefore, variations were related mainly to discrete polymorphisms in the 15 genomic regions tested, such as point mutations, deletions, and insertions, apart from the existence of exclusive genomic segments or rearrangements. Furthermore, seedling health analysis in sugarcane nurseries conducted at Centro de Cana, IAC, reveal that X. albilineans represents the major systemic disease in Brazilian sugarcane fields, occurring silently in its latent form (S. Creste, personal communication).

To gain insight into the aggressiveness and phenotypic variation of the Brazilian X. albilineans, we selected four representative strains with differing virulence capabilities (DVCs) for full-genome sequencing and annotation. Strains Xa04 and Xa11 are considered highly virulent because they lead to acute leaf scald symptoms in the susceptible sugarcane genotype, while Xa21 and Xa26 cause chronic disease symptoms with various intensities (8). A single-point mid-log *in vitro* transcriptome sampling enabled accurate basecalling of the long reads used in each genome assembly. Transcriptomic data also support that the genomes express similarly. We further compared the four strains regarding their *in vitro* growth curve and albicidin production.

The chromosomal organization was conserved when all strains were compared to the previously sequenced X. albilineans strains. Structurally, within colinear regions, no major genomic rearrangements were observed, and only small variations accounted for their genetic diversity. Characteristics that were conserved among all strains included the albicidin biosynthetic cluster, the Salmonella pathogenicity island 1 type 3 secretion system (SPI-1 T3SS), and the recently described Pseudomonas syringae blue-light perception system (9, 13). As few as 91 conserved genes presented single nucleotide polymorphisms (SNPs) that distinguished the Brazilian isolates from the previously described X. albilineans strains. Interestingly, a unique composition of mobile genetic elements (MGEs) was observed that distinguished each isolate and the reference genomes. The *in vitro* transcriptome of each isolate supports that over 75% of the predicted genes are expressed, including the previously described SSR markers. Under *in vitro* growth conditions, our data support that all strains behave similarly under controlled conditions at the gene expression level.

## RESULTS

### Assembly, annotation, and comparative analyses.

The sequencing of the Xa04 and Xa21 isolates generated 11.6 Gb of MinION high-quality data (*N*_50_ read length of 25,830 bp, MinION quality score of 11, average read length of 13,634 bp), while the sequencing of strains Xa11 and Xa26 generated 4.8 gigabases (*N*_50_ read length of 34,300 bp, MinION quality score of 10, average read length of 22,211 bp). Sequencing coverage between strains is presented in Table 1. *De novo* assembly was performed with two different methodologies, strains Xa04 and Xa11 were assembled using the Long Read Support module of the Qiagen CLC Genomics Workbench v.20.0.2 software (https://digitalinsights.qiagen.com/products-overview/discovery-insights-portfolio/analysis-and-visualization/qiagen-clc-genomics-workbench/) (14), while strains Xa21 and Xa26 were assembled with Canu v.2.0 software. The assembled nucleotide sequence was corrected with 6.6 Gb of Illumina HiSeq 1500 *in vitro* transcriptome trimmed data (90% of the bases with quality score >30) using the Long Read Support module of the CLC Genomics Workbench software. All genomes were assembled as circular chromosomes, and no plasmids were identified in the Brazilian X. albilineans isolates (Table 1).

**TABLE 1:** General information about Xanthomonas albilineans strains, including origin, sequencing information, and genome features^*b*^

Characteristic	Data for Xanthomonas albilineans strain:					
	GPE PC73^*b*^	Xa-FJ1	Xa04	Xa11	Xa21	Xa26
Isolation of strains						
Host	H63-1418	YG48	IBSBF-2256	SP90-7027	CV5007 1791	IACSP07-4616
Site of isolation	Roujol, Guadeloupe	Zhangzhou, China	Santa Adélia, São Paulo, Brazil	Conchal, São Paulo, Brazil	Araçatuba, São Paulo, Brazil	Mococa, São Paulo, Brazil
Yr of isolation	2003	2015	2010	2010	2010	2010
Pathogenicity degree			2.05	2.07	1.33	1.63
Symptom scale^*a*^			6.846	6.925	2.819	4.207
Sequencing information						
Sequencing platform	Sanger	PacBio RSII, Illumina PE150	MinION, Illumina^*c*^	MinION, Illumina^*c*^	MinION, Illumina^*c*^	MinION, Illumina^*c*^
Coverage (×)	17	206/570	1,500	417	1,200	499
Size (bp)	3,768,695	3,724,581	3,785,042	3,908,432	3,959,098	3,885,596
%GC content	62.98	63.00	62.98	62.91	62.88	62.97
No. of plasmids	3	1				
Genomic features						
No. of protein-coding sequences	3,115	3,176	3,141	3,255	3,283	3,257
No. of tRNAs	53	53	53	54	54	53
No. of rRNAs	4	4	4	4	4	4
No. of tmRNAs	1	1	1	1	1	1
No. of CRISPR regions	2	2	3	3	3	3
No. of prophages	4	3	3	6	5	5
No. of ISs^*d*^	35	35	40	42	41	35
Reference or source	5	23	This study	This study	This study	This study

a Symptoms scale according to reference 8.

b Used as reference genome for this study.

c Transcriptomic data from Illumina for polishing.

d Complete ISs considered only.

Gene annotation was performed with Prokka v.1.12 software, by which the following gene counts were identified: 3,141 open reading frames (ORFs) in Xa04, 3,255 in Xa11, 3,283 in Xa21, and 3,257 in Xa26. Functional annotation also allowed the identification of two ribosomal (rRNA) gene clusters. tRNAscan-SE predicted 53 tRNAs in the Xa04 and Xa26 genomes as in the reference genomes, while Xa11 and Xa21 have 54 tRNA loci, both carrying an extra tRNA-Ser (CGA). All Brazilian isolates shared with the reference genomes a transfer-messenger RNA (tmRNA) locus organized as a type II toxin-antitoxin system (ratA), SsrA-binding protein (smpB), and tmRNA (ssrA) (15).

**FIG 1:**
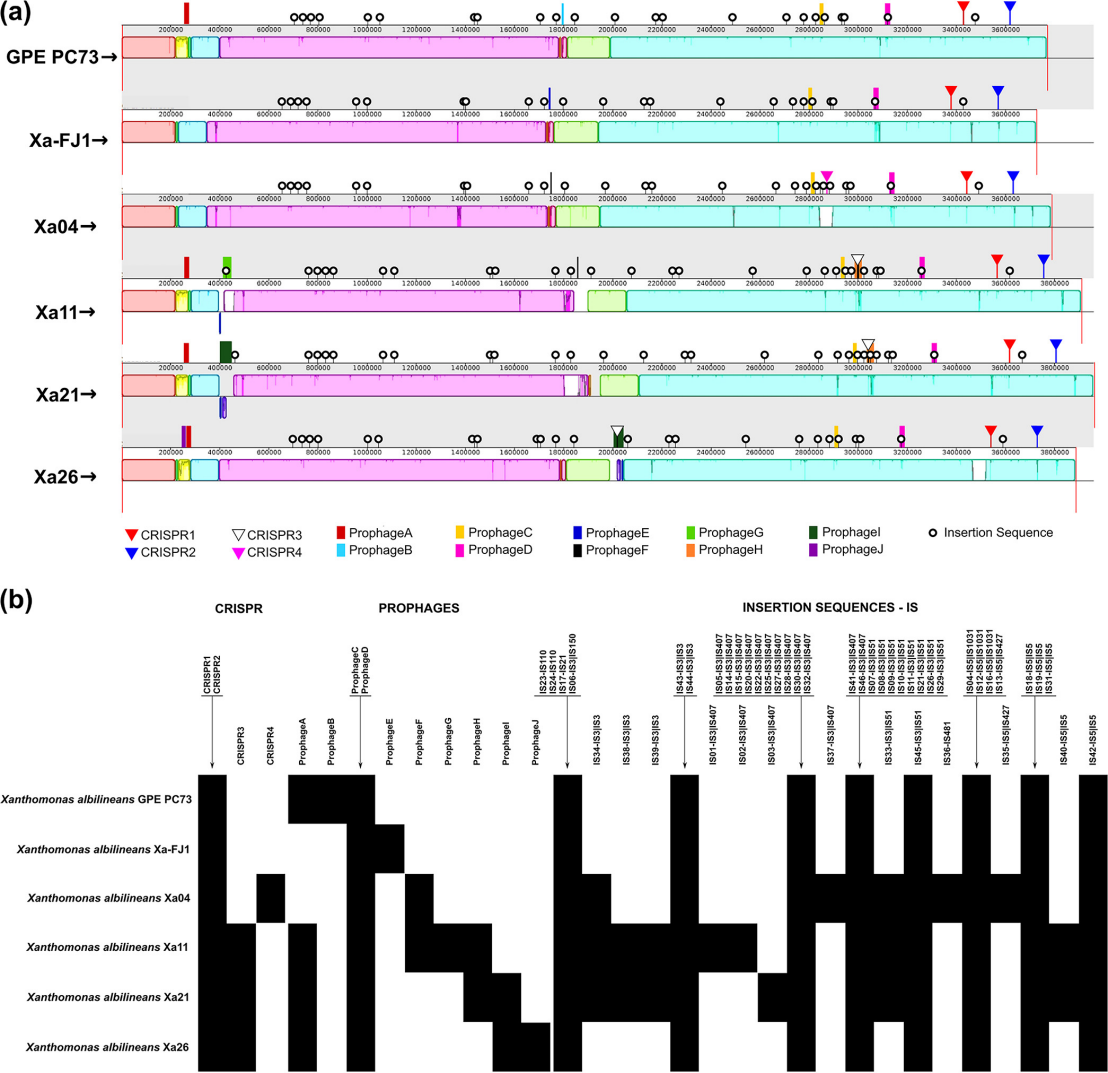
Comparative genomics analyses. (a) Global alignment of X. albilineans genomes with the progressiveMauve tool. From top to bottom are the reference strains GPE PC73 and Xa-FJ1, followed by the Brazilian strains Xa04, Xa11, Xa21, and Xa26. In the image, the sequences start and end with a red vertical bar (open genomes in dnaA). The blocks with similar colors indicate the conserved regions between the genomes; the white regions within the blocks represent sequences with low global similarity, while the white and light gray regions outside the blocks represent unique sequences. (b) Genome features present in each isolate analyzed, including CRISPR regions, prophages, and insertion sequences. Features present in the genomes are marked with black squares (similar genomic context); absences are marked with white squares. IS numbering was defined by position in the genome followed by the family and group associated with the sequence (IS01 to IS3, subgroup IS407).

Comparative genomic analysis was performed from three perspectives, (i) global genome collinearity, (ii) feature composition analyses, and (iii) pan- and core genome evolution analysis. The global genome alignment performed with progressiveMAUVE revealed high similarity between the reference strains and the Brazilian strains of X. albilineans (Fig. 1a), where the absence of large deletions and insertions, as well as the presence of few rearrangements, was observed. The genome collinearity is interspersed with regions of low global similarity and unique sequences (Fig. 1a and b), particularly CRISPR regions and mobile genetic elements.

The six genomes were submitted to CRISPRCasFinder (16), which identified the existence of three CRISPR regions in each of the Brazilian strain genomes, two of which (CRISPR1 and CRISPR2) are present in all genomes, including GPE PC73 and Xa-FJ1, and located at the same conserved region (Fig. 1a). These two correspond to complete CRISPR loci from type I Cas encoding proteins. CRISPR1 is type I-C, and CRISPR2 is type I-F. CRISPR3 is present in the Xa11, Xa21, and Xa26 genomes within prophage regions prophage H and prophage I, respectively. CRISPR4 is also an incomplete version of a CRISPR locus, is unique to Xa04, and is located at the same insertion sequence (IS)-rich region as CRISPR3 from Xa11 and Xa21. Both CRISPR3 and CRISPR4 have HNH endonuclease upstream spacer and repeat regions. Of note is that CRISPR3 and CRISPR4 repeats are identical to the CRISPR2 repeat nucleotide sequence, while some spacers are shared, and others are unique to the genome. Brazilian strains have a greater number of CRISPR spacers than the two reference strains (Fig. 1b).

Prediction of the mobilome gene set was performed using ISsaga (17) and PHASTER (18, 19), with subsequent manual inspection to define the insertion sequences and prophages, respectively. The IS copy number varied between the genomes. In the reference strains and the Xa26 strain, 35 ISs were annotated, while in the Xa04, Xa11, and Xa21 strains, the software identified 40, 42, and 41 ISs, respectively. These ISs were characterized as belonging to 5 families and 10 subgroups, namely, IS110; IS21; IS3, subgroups IS150, IS3, IS407, and IS51; IS481; and IS5, subgroups IS1027, IS427, and IS5. The IS51 and IS407 subgroups, belonging to the IS3 family, were the most prevalent ISs, with at least nine insertions in each genome. Another important observation is the existence of a unique IS481 identified only in the Xa04 strain (Fig. 1b).

We comprehensively characterized a 1,320-bp IS element named ISXal1 that was present in equal numbers of copies (6) and sites of insertion in GPE PC73, Xa04, Xa11, and Xa21, whereas they were distributed in 11 and 8 copies in the genomes of Xa-FJ1 and Xa26, respectively. This element belongs to the IS45, IS3, and IS51 families with a perfect 27-bp-long inverted repeat (IR) and two ORFs. The first presents an in-frame stop codon, but a ribosomal −1 frameshift between the two coding regions during expression might result in a functional DDE transposase. However, four of the six copies shared by all X. albilineans are flanked by perfect direct repeats. ISXal1 presents more than 90% of nucleotide identity along its nucleotide sequence compared with a prospected IS element in the genome of *Variovorax* sp. strain WDL1 and with ISStma17 of Stenotrophomonas maltophilia, including identical 3′ IRs to both of them. This element is also shared with many species of *Xanthomonas* but with slightly lower nucleotide identity. Altogether, these analyses lead us to propose that ISXal1 can still be an active element or was until recently.

A total of 10 different prophages were identified in the 6 X. albilineans genomes, each with a unique infection history. These are named prophages A to J and are not equally distributed across all isolates; for instance, the Xa-FJ1 and Xa04 strains have three prophage loci, and the GPE PC73 strain has four prophage loci, while the Xa21 and Xa26 strains have five, and Xa11 has six. Two of these prophages (prophage C and prophage D) are common to all genomes and sit at the same location, including the references; thus, they are ancestral. Four are common to subsets: prophage A, common to GPE PC73, Xa11, Xa21, and Xa26, is located in a fragment that is unique to these strains; prophage F is found in isolates considered highly pathogenic (Xa04 and Xa11), but their genomic locations are distinct; prophage H, common to Xa11 and Xa21, is collocated with CRIPR3 in a region rich in mobile genetic elements; and prophage I, containing at least six phage-related genes, is common to strains considered less pathogenic (Xa21 and Xa26) but located in different genomic regions and orientations. Four unique prophages were also identified in the GPE PC73, Xa-FJ1, Xa11, and Xa26 genomes, namely, prophage B, prophage E, prophage G, and prophage J, respectively. Prophages B and E are unique to GPE PC73 and Xa-FJ1, respectively, but are located in the same region, and prophage G is located in the same region as Xa21.

A 51-kb region containing prophage A (yellow block in Fig. 1a) harbors six phage-related genes, more than 50 ORFs of unknown function, and four DNA methyltransferases. The latter might be involved in controlling the expression of a number of virulence genes, favoring the establishment of different phenotypes among the strains, as previously reported for various bacteria (20). The region is accompanied by an 11.5-kb fragment (green block in Fig. 1a) located up- or downstream and shared among all X. albilineans, which encompasses, among other genes, a tyrosine-type recombinase/integrase and two tRNA-associated genes. Those might have served as hot spots for chromosomal rearrangement or phage integration within the genome.

**FIG 2:**
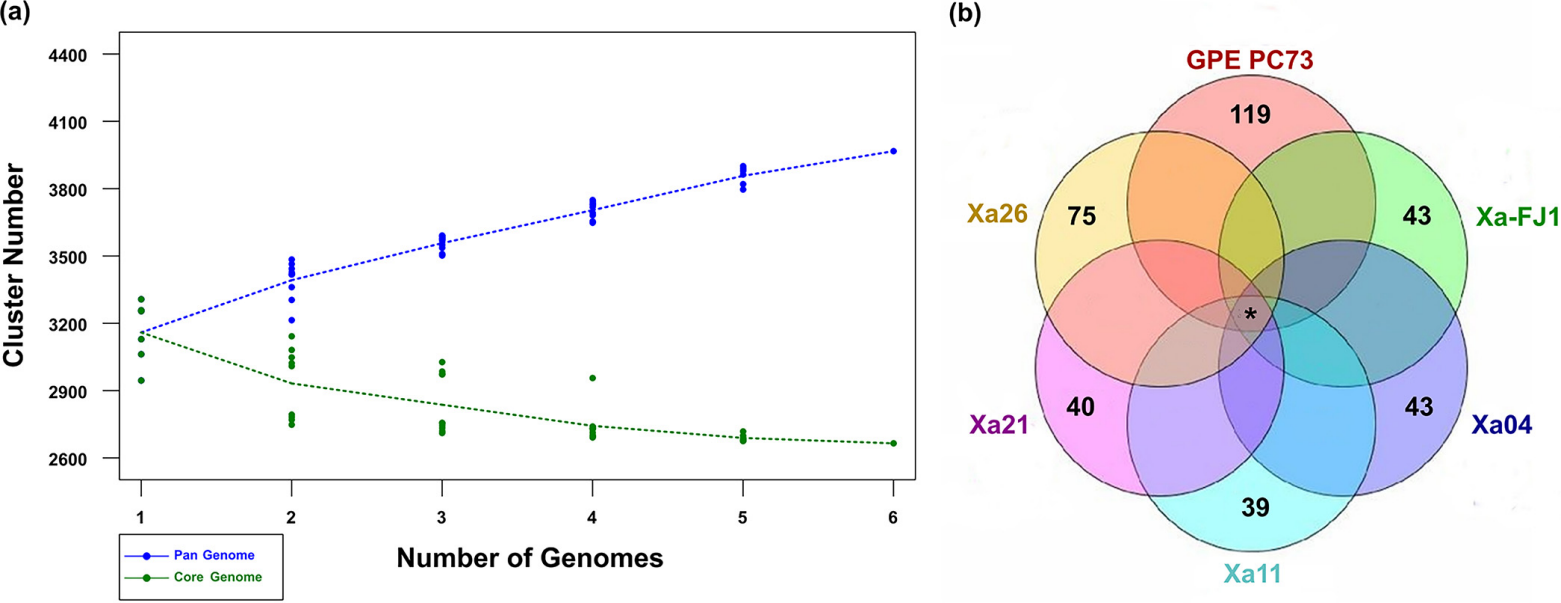
Pan- and core genome identification. (a) Pan- and core genome evolution of X. albilineans. The inclusion of new genomes in the analysis shows an increase in the pangenome size (blue line) and a reduction in the core genome size (green line). The cluster number calculation was randomized for 6 sets, each one represented by a single point. (b) Results of the first clustering iteration organized graphically in a Venn diagram. In the center, the number of ortholog groups (orthogroups) shared by all X. albilineans complete genomes, showing core genome; at the ends are the numbers of unique genes present in each genome (singletons). *, 2,716 shared genes. The number of ortholog groups shared by subsets of genomes is not presented.

The core and pangenome analyses indicated that the inclusion of new genome sequences increased the pangenome size and reduced the core genome size (Fig. 2a). These results support that X. albilineans is an “open” and flexible genome, and its complete gene repertoire is not yet fully known. A clustering iteration with OrthoMCL (21) was used to identify the core and variable genomes. We considered the core genome to be the orthologous genes common to all completely assembled genomes and the variable genome to be the orthologous genes common to subsets of those genomes and unique genes (singletons). After processing, 3,212 ortholog groups were identified, of which 2,716 were common to all genomes, and 496 were common to subsets of them. In addition, 359 unique genes were identified among the 6 analyzed genomes (Fig. 2b). Among the 2,716 ortholog groups belonging to the core genome, 97 consisted of multicopy genes and 2,619 of single-copy genes. A total of 2,443 genes were identical (100% identity and 100% nucleotide coverage) in the single-copy gene subset and 176 similar genes, of which 70 had SNPs and 106 had indels between the sequences.

Interestingly, several genes associated with pathogenicity, such as adhesion genes, cell wall-degrading enzymes, and polysaccharide synthesis, together with genes involved in secretion systems, nonribosomal peptide synthetase (NRPS), diffusible signal factor (DSF), and the flagellar operon, are amid the core genome collection. An exception is the TonB receptor gene family, which is highly diverse.

### Evolutionary and genetic diversity.

**FIG 3:**
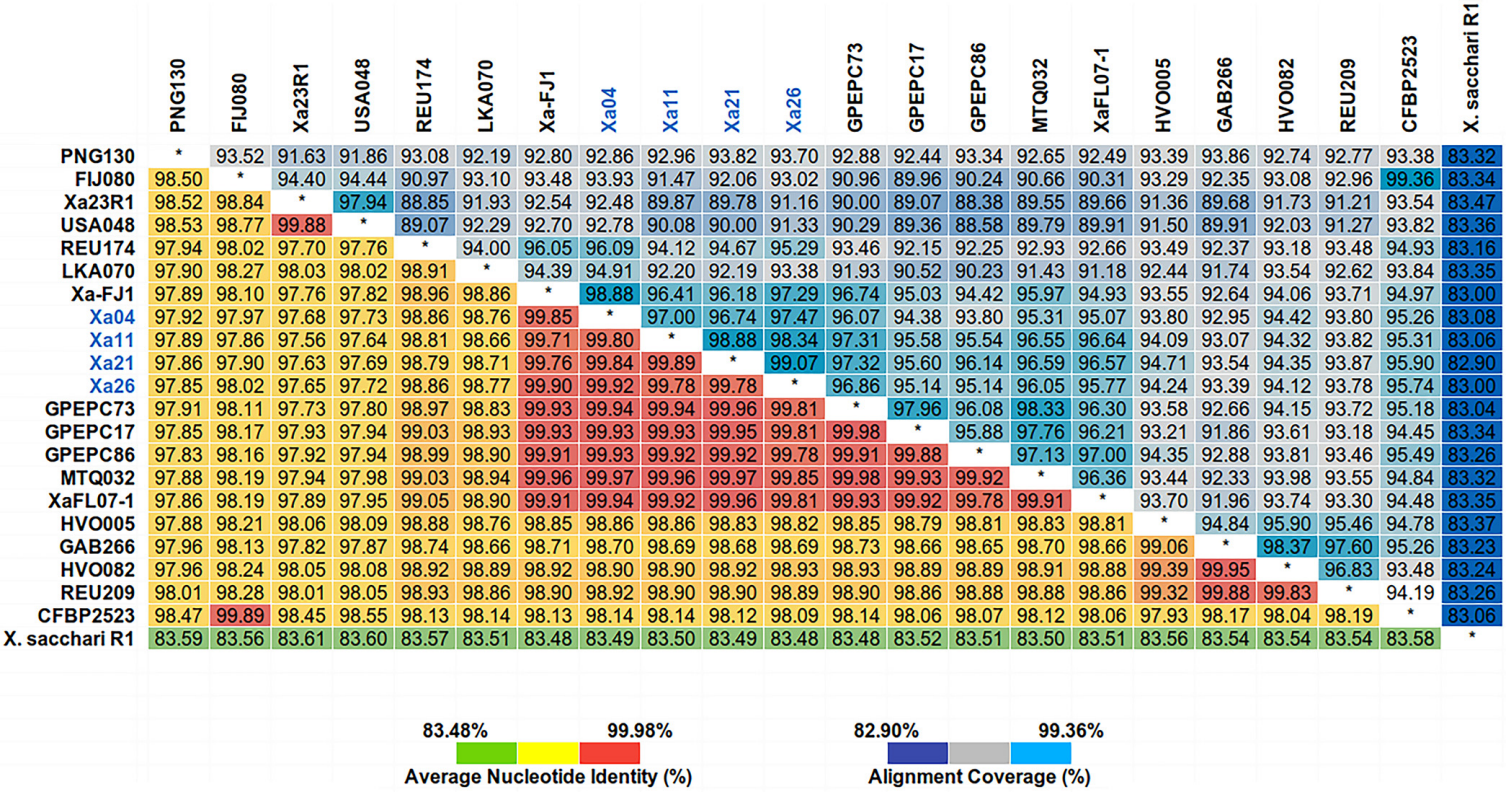
Heatmap of average nucleotide identity (ANI) and alignment coverage based on the total genome sequence of 21 strains of X. albilineans and 1 X. sacchari strain (outgroup). Brazilian strains are marked in blue. ANI values (%) for each two genome comparisons can be seen in the lower triangle of the matrix. The alignment coverage values (%) are arranged in the upper triangle.

Based on a second OrthoMCL clustering iteration to determine the gene set shared between the 4 Brazilian strains and the 17 public strains of X. albilineans, as well as the selected outgroup R1 strain of X. sacchari (22) (see Table S1 in the supplemental material), a common gene set of 91 genes across the strains and X. sacchari were identified (Table S3). The average nucleotide identity (ANI) calculation was based on the total genome sequence of all X. albilineans strains and the X. sacchari R1 strain (Fig. 3). The values ranged from 83.48 to 99.98% among all genomes analyzed, whereas, for X. albilineans, ANI values ranged from 97.56 to 99.98%. In addition, the X. albilineans strains share 83.48 to 83.61% ANI with strain R1 of X. sacchari. The Brazilian strains (≥99.78%), together with the Guadeloupe Island strains (GPE PC73, GPE PC17, and GPE PC86) (ANI values, 99.81 to 99.96%, 99.81 to 99.95%, and 99.78 to 99.93%, respectively), the Martinique Island strain (MTQ032) (ANI values, 99.85 to 99.97%), one strain from the United States (XaFL07-1) (ANI values, 99.81 to 99.96%), and the Chinese strain (Xa-FJ1) (ANI values, 99.71 to 99.90%), shared higher ANI values. The less similar strains to the Brazilian strains are PNG130 (ANI values, 97.85 to 97.92%), FIJ080 (ANI values, 97.96 to 98.02%), CFBP2523 (ANI values, 98.09 to 98.14%), and two U.S. strains (Xa23R1 ANI values, 97.56 to 97.68%, and U.S. 048 ANI values, 97.64 to 97.73%, respectively).

**FIG 4:**
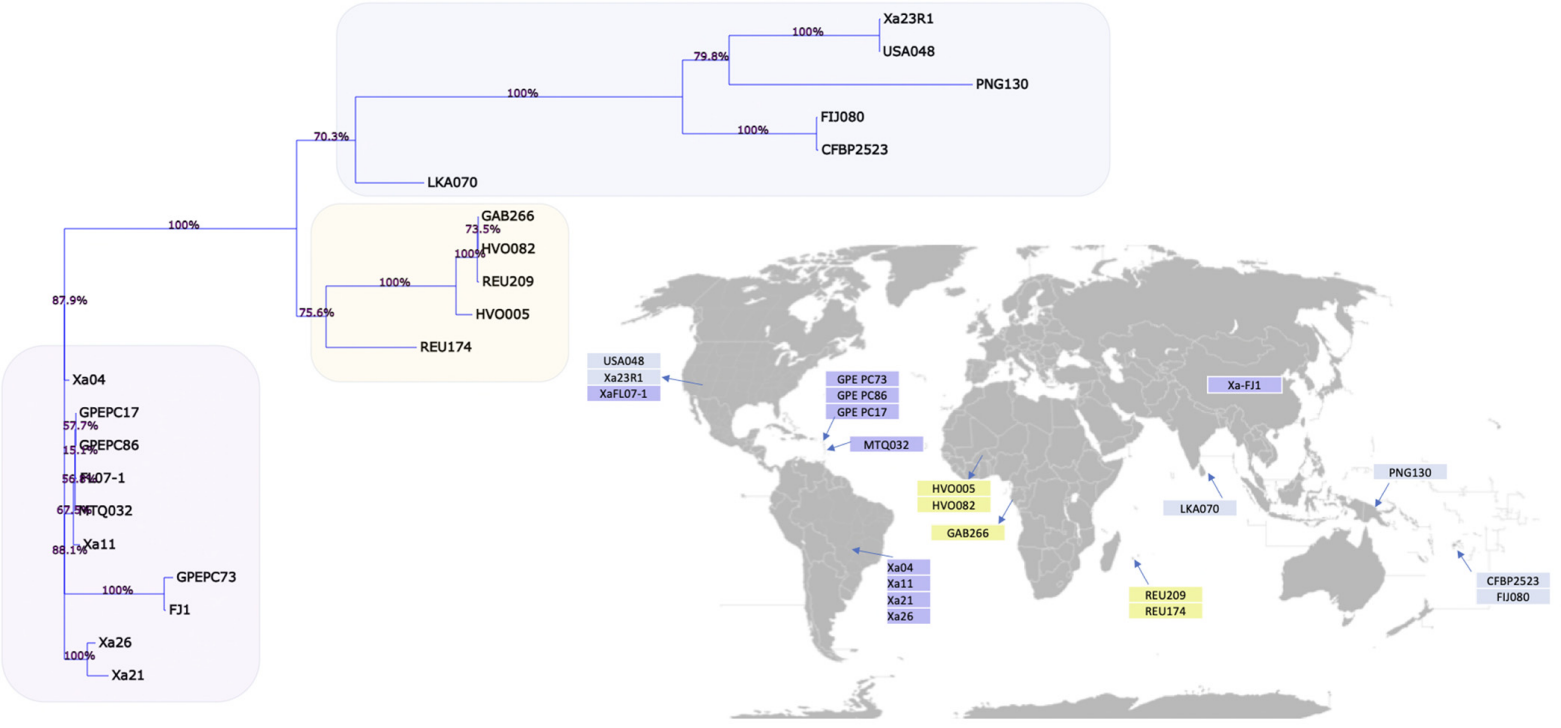
Phylogenetic tree of 21 X. albilineans strains. BioNJ tree topology is presented with bootstrap values indicated on top of the branches. Strains are mapped to countries following data collection as described in Table S1 in the supplemental material. Strain names appear in colored boxes matching tree topology inference. The worldmap can be found at the link https://upload.wikimedia.org/wikipedia/commons/4/47/Mapa_mundi_divisiones_blanco.PNG, and it is nested under the Wikimedia initiative (https://www.wikimedia.org/).

Genetic diversity within an informative sample of the 91 genes previously described was also examined among the 21 X. albilineans strains over 67,654 nucleotide sites, of which 65,796 (97.25%) are conserved and did not present any polymorphism. Figure 4 presents the resulting phylogenetic tree associated with the strain sampling locality. First, tree branches reveal a geographical emerging pattern with Asiatic, African, and American strains clustering each together, with the exception of Xa23R1 (U.S.), USA048 (U.S.), and XaFJ1 (Chinese) strains. Within the American clade, the Brazilian strains fall in two distinct branches, both poorly supported. The branch lengths of the Brazilian isolates, as well as the American strains, are exceedingly short, which indicates a close genetic relationship with the neighboring lineages but also prevents further resolution of the tree due to a lack of phylogenetically informative signals.

### *In vitro* transcriptomics.

As described above, we found that, to some extent, their genomes do not echo differences from the most to least aggressive Brazilian X. albilineans strains. Additionally, for the genomes of the most pathogenic isolate group, with a corresponding less pathogenic isolate as duplet, Xa04 groups with Xa26 and Xa11 with Xa21. To further investigate, a transcriptomic approach was undertaken in which the four strains were grown to mid-log phase under *in vitro* conditions to explore whether differences in expression under controlled conditions could underlie differences in gene expression capabilities.

The total RNA transcriptome of the four X. albilineans strains grown *in vitro* (see conditions described below) yielded together over 29 million reads. After filtering and trimming, they resulted in 6.3, 7.2, 8.2, and 7.2 million paired-end (PE) reads and 487, 562, 638, and 557 Mbp of Xa04, Xa11, Xa21, and Xa26, respectively. Overall, 99.98% of the bases had PHRED quality scores higher than 20, and 94% had scores higher than 30. When mapped onto their respective genomes, the distance between 90% of the paired reads varied from 60 to 200 bp, in agreement with the fragment size selection of the cDNA selected prior to sequencing.

Compared with the references, approximately 75% of the PE reads were mapped onto annotated ORFs, 10% were mapped onto regions not assigned as coding genes, 0.3% were mapped onto the ribosomal DNA gene cluster, and 14.7% were not mapped anywhere, mainly because, according to our parameters, unpaired reads were not counted. Based on read mapping, the ratio of total RNA (excluding rRNA) to rRNA of all sequences was 280:1, supporting that our in-house rRNA depletion protocol and probes worked very efficiently. Most of the annotated ORFs are supported by reads mapped against them, adding biological evidence to those. Only a few genes did not exhibit some level of expression *in vitro*, namely, 19, 23, 35, and 40 ORFs from a total of 3,188, 3,312, 3,365, and 3,316 predicted genes in the genomes of Xa04, Xa11, Xa21, and Xa26, respectively. Most of the insubstantial ORFs corresponded to hypothetical proteins or faulty tRNA predictions and the SSR IACXa09, which is predicted to encode an autotransporter serine protease.

### Using ribosomal proteins to assess differences in transcriptome and TPM values.

**FIG 5:**
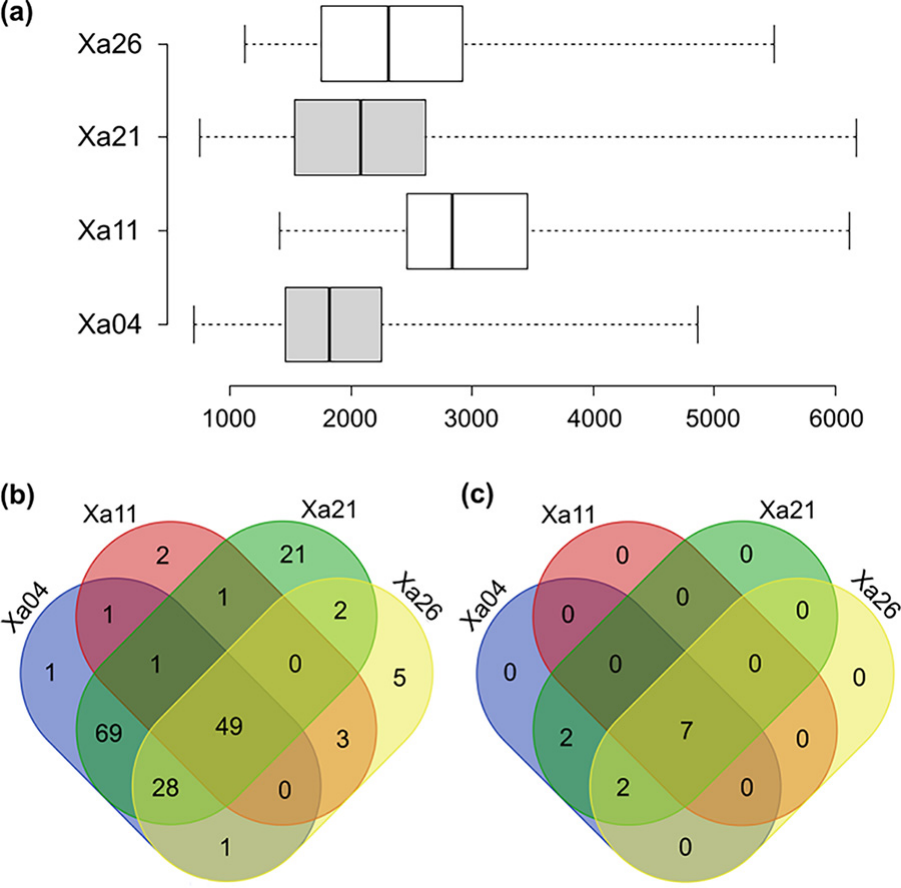
Transcriptome analysis and Venn diagram. (a) Center lines show the medians, box limits indicate the 25th and 75th percentiles as determined by R software, and whiskers extend to minimum and maximum values. *n* = 54 sample points. (b) Venn diagram showing the shared genes among the four Brazilian X. albilineans strains for which TPM values fall in the range of the ribosomal protein set. Genes in the range and above ribosomal protein TPM values are included (Table S3 in the supplemental material). (c) Venn diagram showing the shared genes among the four Brazilian X. albilineans strains for which TPM values are above those of ribosomal proteins, supporting very high expression in the cell. Included are genes above ribosomal protein TPM values (Table S3).

As these strains show little genomic differences and most of the annotated genes had mapped reads, we used the transcripts per million (TPM) values of ribosomal proteins (RPs) to assess the efficacy of the transcriptome profile among the strains. The rationale undertaken is that this set of 54 ribosomal protein-coding genes would present similar TPM values since they are single copy, constitutive, and nearly identical among the four strains and because the cells were all grown *in vitro* under the same conditions and harvested in the log growth phase for the transcriptome experiment. Both the median and variance of TPM values for this set of proteins fall within a comparable range (Fig. 5a).

Ribosomal proteins are canonically found to be highly expressed in cells. Using ribosomal protein as a proxy for high gene expression, we ranked all chromosomal genes by their TPM values and further identified genes for which TPM values fall in the range of the ribosomal protein set and therefore infer significant expression in the cell. The ranking was performed independently for each X. albilineans strain, and we identified 49 genes with high expression (Fig. 5b and Table S3). Among those genes, there are five hypothetical proteins (ortholog groups [orthogroups] 179, 886, 1576, 1778, and 2646). Orthogroups 1576 and 1778 are among the most highly expressed genes in the transcriptome compared to ribosomal protein-encoding genes.

Additionally, we also determined which genes could be considered to have very high expression by determining the genes for which TPM values are higher than the highest ribosomal protein TPM value. Seven genes were common to all four strains, a DNA binding protein; three members of the large outer membrane protein (OMP) family, orthogroup 480 (*ompA*), orthogroup 2140 (Ax21), and orthogroup 2036 (outer membrane beta-barrel protein); orthogroup 872 (*cspA*); and two hypothetical proteins (orthogroup 1576 and 1778) previously mentioned (Fig. 5c and Table S3).

### T3SS and X. albilineans 1 gene clusters.

**FIG 6:**
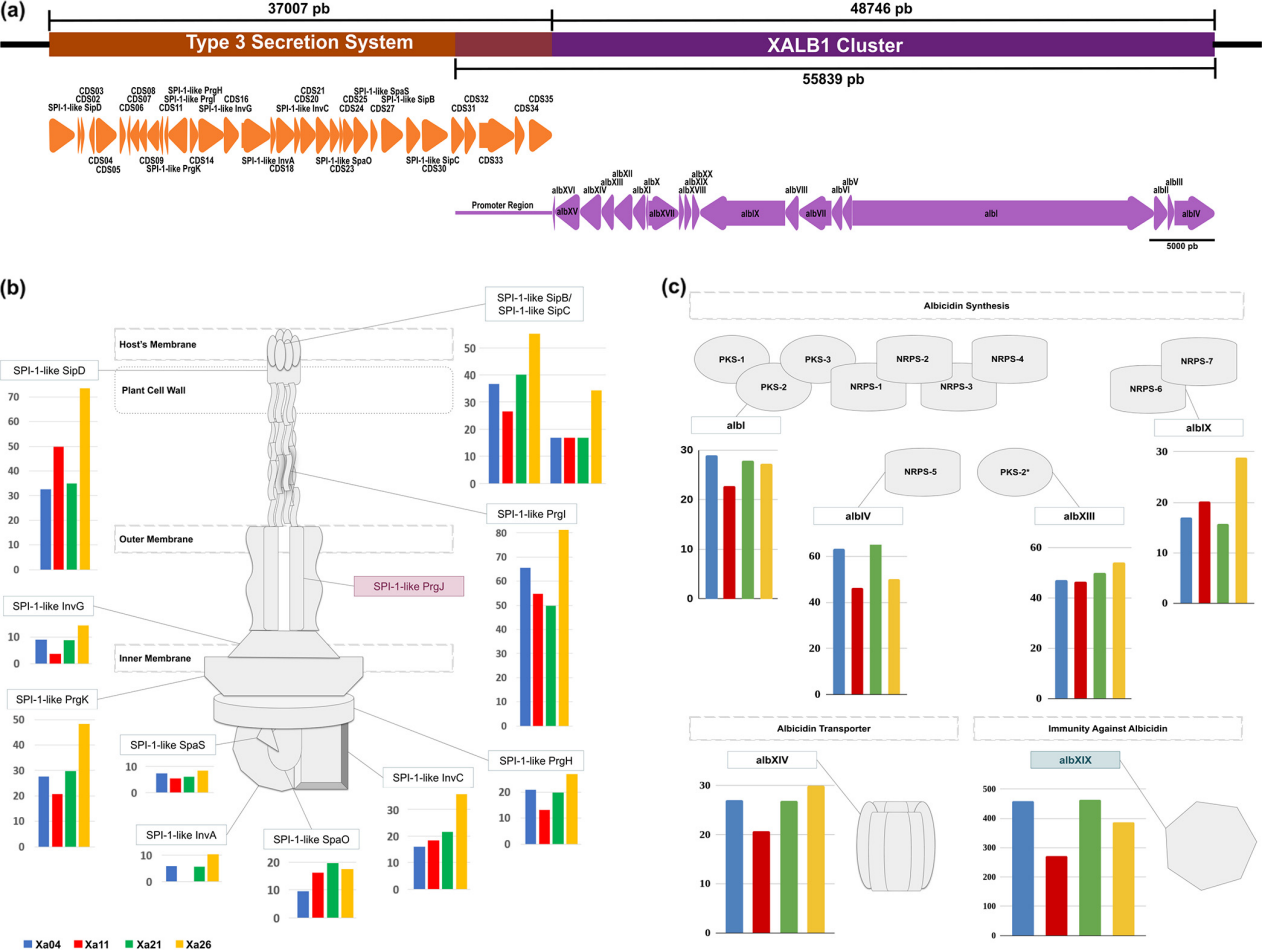
SPI-1 T3SS and the X. albilineans 1 cluster analysis. (a) Representation of the genomic region containing the SPI-1 T3SS (orange) and the X. albilineans 1 cluster (purple); the image is plotted proportionally. The overlap region between the structures is marked by the overlap of orange and purple boxes. The orange arrows represent the SPI-1 T3SS ORFs, while the purple arrows represent the X. albilineans 1 cluster ORFs. The arrow direction indicates the ORF direction within the genomes. (b) Gene expression profile of SPI-1 T3SS structural proteins from the four Brazilian strains. The most aggressive strains, Xa04 (blue) and Xa11 (red), are plotted on the left, while the less aggressive strains, Xa21 (green) and Xa26 (yellow), are plotted on the right. Structural genes not identified in the SPI-1 T3SS are marked in red. (c) Gene expression profile from the four Brazilian strains of synthesis and resistance genes to albicidin phytotoxin (representation shows the PKS and NRPS modules encoded by each synthesis-related ORF). The albXIX gene with the highest TPM among all genes in the X. albilineans 1 cluster is marked in light blue. The TPM bars presented in panels b and c are only used as a reference supporting similar expression across the strains.

Unlike other genus members, X. albilineans has some unusual genomic features. The most relevant are the absence of hypersensitive response and pathogenicity type III secretion system (Hrp-T3SS), the presence of Salmonella pathogenicity island 1 type III secretion system (SPI-1 T3SS), and the ability to produce the phytotoxin albicidin from the X. albilineans 1 gene cluster (9, 23). The analysis of these structures showed that SPI-1 T3SS and X. albilineans 1 are adjacent to each other and have an overlapping region, a sequence of 7,093 bp that encodes coding sequences (CDSs) 31 to 35 of the SPI-1 T3SS and also corresponds to the promoter region of the X. albilineans 1 cluster (Fig. 6a).

The gene expression profiles of the T3SS structural proteins from the four Brazilian strains were similar and low, as the highest expression level was just below 90 TPM and corresponded to the gene that encodes the SPI-1-like PrgI protein, which, in multiple copies, forms the pilus. Considering that the samples were collected from *in vitro* bacterial suspension cultures, future experiments from an *in planta* sampling could provide insights into the relevance of the T3SS in the disease establishment. Despite low expression levels compared to ribosomal proteins, most of the genes from the T3SS cluster are expressed (Fig. 6b), suggesting that the apparatus may also contribute to the *in vitro* growth survival. T3SS from Xa11 displays the lowest expression in addition to being the only one that presents null expression of T3SS-CDS17 (SPI-1-like invA, an export apparatus protein composing the gate) and T3SS-CDS33 (a yet-uncharacterized protein, with low similarity to a hydrolase).

Expression of the X. albilineans 1 gene cluster *in vitro* is supported in all four Brazilian isolates (Fig. 6c), with TPM values ranging from null expression to 465. Genes responsible for synthesis (*albI*, *albIV*, *albIX*, and *albXIII*) and resistance to albicidin (*albXIV* and *albXIX*) are actively transcribed in the bacterial cells, suggesting that the phytotoxin is synthesized under *in vitro* growth conditions (Fig. 6c). The *albXIX* gene, which encodes an immunity protein against albicidin, is expressed in all strains, as is albicidin synthesis supported by the inhibition ring text described below. The TPM bars presented in Fig. 6b and c are only used as a reference supporting similar expression across the strains.

### *In vitro* growth studies and albicidin production.

**FIG 7:**
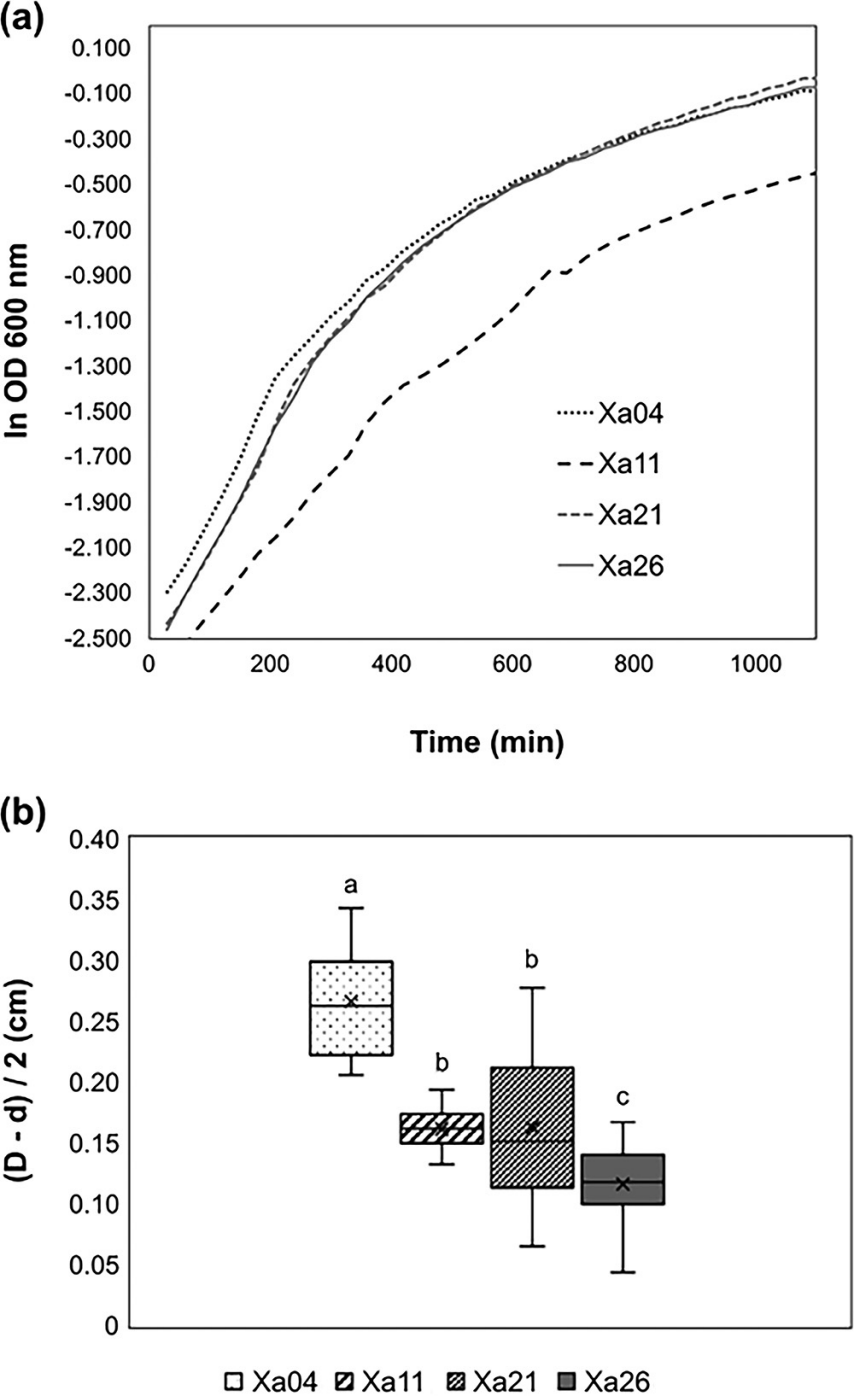
Growth curves and albicidin. (a) Growth curve of four Brazilian strains of X. albilineans. (b) Albicidin inhibition ring of four Brazilian strains of X. albilineans in E. coli. The inhibition ring is given by *D* (diameter of the E. coli growth inhibition halo) minus *d* (diameter of the X. albilineans culture).

The Brazilian strains were classified into four pathotypes, based on a symptom rating scale after inoculation of a susceptible sugarcane genotype growing at a greenhouse (8). To expand knowledge into the biology of those strains, we defined their growth rates and growth curve (Fig. 7a). Xa11, the most aggressive strain, presented the lowest growth rate, whereas the Xa04 growth rate was close to the growth rates of Xa21 and Xa26, the less aggressive strains (8), indicating that for X. albilineans, the *in vitro* growth rate does not correlate with aggressiveness.

We further inspected the *in vitro* albicidin production by testing the inhibition ring formed when Escherichia coli was inoculated in X. albilineans cultures (12). Xa04 has the highest values for inhibition rings, while Xa11 values are similar to Xa21. A one-way analysis of variance (ANOVA) test followed by Tukey’s test grouped groups the strains into three classes, a (Xa04), b (Xa21 and Xa11), and c (Xa26) (Fig. 7b). Interestingly, the aggressive strains (Xa04 and Xa11) produce dissimilar inhibition rings, suggesting that albicidin production is different (Fig. 7b). Xa11 and Xa21, with similar genomes, are clustered together with regard to the inhibition ring test. However, Xa04 and Xa26, which also share similar genome structures, have the most distant inhibition ring test values, Xa04 is the second most aggressive, and Xa26 is intermediate between the most aggressive ones and Xa21. Our results do not correlate aggressiveness with the albicidin inhibition ring, supporting previous studies (12). Despite no correlation being observed, we found significant differences between strains regarding E. coli inhibition of halo production.

## DISCUSSION

Comparative genomics has been used for the last 2 decades as a powerful tool to gain insight into microbial genomes, especially to understand the genome variation related to niche occupation, be it a pathogen, a symbiont, or a specialized environmental adaptation. The present work describes the sequencing of four X. albilineans isolates recovered from sugarcane cultivars grown in Brazilian fields with differing leaf scald symptoms. The genome structure is highly conserved between isolates as well as compared to the other previously sequenced strains. The mobile portion revealed some unique features, such as how prophage F, which harbors a ZOT-like encoding gene, is present in the most virulent strains. The ZOT-like encoding gene is also described in XFJ1. Prophage I is shared by the two least virulent strains, and prophage G is present only in Xa11. Several unique genes are present in these prophage regions, most of which are hypothetical. In addition, the study of the CRISPR regions revealed that the Brazilian isolates were subjected to unique bacteriophage infections that resulted in the addition of unique spacer regions. Only CRISPR1 and CRISPR2 possess complete type 1 Cas-containing loci. Interestingly, CRISPR3 and CRISPR4 contain repeats identical to CRISPR2 but are located in different genomic environments with a predicted HNH endonuclease upstream of the initial repeat. CRISPR3, present in the Xa11, Xa21, and Xa26 genomes, is located at the same genomic position in the first two isolates but not in Xa26. This specific region from Xa11 and Xa21 is highly populated with insertion sequences (ISs). CRISPR4 is an acquisition unique to Xa04, and it is located in the same IS-rich region as CRISPR3. In conclusion, MGE and CRISPR regions provide genomic differentiation to these bacterial strains.

Pangenome evolution analysis allowed identification of gene diversity through the determination of the core genome, formed by genes present in all strains analyzed, and the variable genome, formed by genes absent in one or more strains (accessory genome), in addition to genes that are limited to a particular isolate (24, 25). In bacterial genomes, the set of exclusive genes can vary from 20% to 40% of the pangenome. This set is responsible for genome flexibility and is often described as essential for pathogenicity, interaction with the host, resistance to drugs, adaptation to specific niches, and other phenotypes of great medical, economic, and industrial importance (25, 26). The X. albilineans genome is “open”; that is, the incorporation of new genomic sequences in the analysis increases the pangenome size (total set of genes, core and variable genomes) and reduces the core genome size (set of genes shared by all genomes), indicating that the complete gene repertoire of the species is not yet fully known. It is necessary to include a greater number of genomes to obtain the complete pangenome of the species (26, 27). The phylogenomic tree and ANI analyses are concordant with previous analyses based on pulsed-field gel electrophoresis (PFGE) and SSR (6, 28). ANI based on the whole genome supports that all sequenced strains are a single species (>95%), with the exception of X. sacchari. However, a slight differentiation in the ANI values can be observed that is also supported by the phylogenomic tree where genetic diversity can be grouped based on their geographical origins (American, African, or Asiatic), with the exceptions of Xa23R1 (United States) and USA048 (United States) that cluster with the Asiatic strains and XaFJ1 (China) that clusters with the American strains close to GPE PC73. The American strains seem to have less genetic variation over time, as the MTQ032 strain was isolated in 1932, while Xa11 and Xa21 Brazilian isolates were recovered from different sugarcane plantations in 2010 (8), and GPE PC73 was isolated in 2003 (11). With the addition of complete genome sequences of these and other *Xanthomonas* strains, novel approaches will enable the understanding of the evolutive forces driving the genetic diversification in these bacterial populations.

Bacterial secretion systems are not only important means of communication and competition between bacteria and interaction with their surroundings but also valuable tools for pathogenicity (29). In addition to participating in the formation of mobility and adhesion-related structures, these systems allow the secretion of various enzymes, toxins, and effectors, essential to inter- and intraspecies competition and the invasion of host target cells (30). In particular, the type III secretion system (T3SS) primarily plays the role of an effector delivery system, which involves mainly suppressing the host immune system and promoting nutrient acquisition (31). X. albilineans, contrary to the other representatives of its genus, lacks an Hrp-T3SS but presents a T3SS that is closely related to SPI-1, which is near the terminus of the replication site and possibly a result of a horizontal gene transfer event (6). The SPI-1 T3SS cluster was not correlated with plant colonization or virulence, once it was absent in at least one pathogenic strain of X. albilineans and because knockout mutants showed no impairment in the promotion of the disease (9). The predicted effector region of the T3SS cluster is similar to toxins predicted to interact with animals. This observation and the fact that T3SS from Salmonella is predicted to have a shorter needle, as expected for bacteria-animal cell interaction, may suggest that it is involved in the association with potential insect vectors when human-driven spread is not the main form of propagation of this pathogen in sugarcane or other hosts.

Not previously described is the genomic position of the X. albilineans 1 cluster, which is head to tail with the T3SS close to the terminus of the bacterial chromosome, as presented in Fig. 6a. These gene clusters are superposed and expressed under *in vitro* conditions. The region described previously (32) as the promoter region is, in fact, the effector containing the region of the T3SS cluster. The first six genes from the X. albilineans 1 cluster are in opposite orientations; thus, it does not support that the previously described promoter region is, in fact, the promoter region of X. albilineans 1. Albicidin, a nonribosomal peptide synthetase (NRPS) toxin, synthesized by the X. albilineans N1 cluster, is a potent DNA gyrase inhibitor that prevents DNA replication of bacteriophages, bacteria, and plastids in plant cells (4, 33, 34). Albicidin is largely accepted as the major contributor to the leaf phenotype but is not a virulence determinant, as GPE PC73 produces symptoms but does not produce albicidin (12). Our results add that the cluster is highly conserved across distinct isolates; however, albicidin production varies among Brazilian isolates and does not correlate with virulence, as the most virulent isolates (Xa04 and Xa11) have significantly different inhibitory ring sizes. Recent results from Kortright et al. (35) demonstrate the use of albicidin as an antibiotic, which could confer an advantage to the X. albilineans bacterial population inside the plant tissues by inhibiting the growth of endophytes.

In pairs, Xa04 and Xa26, as well as Xa11 and Xa21, share high synteny. However, distinct phage infections are shared between the most virulent (Xa04 with Xa11) or least virulent (Xa21 with Xa26) bacterial strains, which unveils an interesting bacteria-phage dynamic in the X. albilineans strain differentiation. *In vitro* transcriptomics further contributed to the subtle differences among the Brazilian isolates, narrowing to a few candidate genes for functional studies. The novelty in the study presented here lies in the systems approach to combine genomics, transcriptomics, and biology of the leaf scald disease agent. Understanding bacterial population dynamics in an infected plant or field is required to better manage Xanthomonas albilineans’s interactions with sugarcane and other plants. Novel approaches are needed to solve this unique interaction.

## MATERIALS AND METHODS

### Bacterial strains.

Four Brazilian isolates of X. albilineans belonging to the Xanthomonas albilineans culture collection of Centro de Cana, IAC, Brazil, were used in this study (SisGeen A6C6170). These organisms were obtained from different sugarcane plantation areas in the state of São Paulo and selected according to their aggressiveness (8). Among these organisms, the two most virulent isolates (Xa04 and Xa11), an intermediate isolate (Xa26), and a low-virulence isolate (Xa21) were selected (8). Biological samples were collected, analyzed for their pathogenicity, and provided by researchers from the Centro da Cana, Campinas Agronomic Institute (IAC), in Ribeirão Preto, São Paulo. GPE PC73 (GenBank accession number FP565176) and Xa-FJ1 (GenBank accession number CP046570) isolates, available in public databases, were adopted as reference genomes for comparative analyses (5, 23).

### *In vitro* bacterial growth assessment.

Bacteria were cultivated in modified Wilbrink’s (MW) medium (sucrose,10 g/L; tryptone, 5 g/L; K_2_HPO_4_·3H_2_O, 0.50 g/L; MgSO_4_ 7H_2_O, 0.25 g/L; Na_2_SO_3_, 0.05 g/L; and distilled water, and pH was adjusted to 6.8 to 7.0) (36) in solid media (agar, 15 g/L) for 4 days at 28°C. Cells were then transferred to 10 mL of MW liquid medium and incubated for 41 h with agitation at 28°C. The optical density at 600 nm (OD_600_) was approximately 1.2, and 60 μL was transferred to a new medium with a volume of 60 mL and incubated for 23 h until the OD_600_ values reached approximately 0.3 to 0.4. The inoculum was centrifuged and resuspended to an OD_600_ of 0.1 and diluted ×10. Finally, 200 μL of inoculum was grown in 96-well plates for 25 h with agitation at 28°C, and the OD_600_ was read at 30-minute intervals in a microplate reader. Growth rates were calculated using GrowthRates software (37).

### Albicidin inhibition assay.

Albicidin production was evaluated by measuring X. albilineans inhibition of Escherichia coli growth. For that purpose, 3 μL of culture of X. albilineans (OD_600_ = 0.3) was spotted in a petri dish with 25 mL of MW medium and incubated for 48 h at 28°C. Then, plates were overlaid with 10 mL LB soft agar (0.7%) with 100 μL E. coli strain DH5α (OD_600_ = 1) and incubated for 48 h at 28°C. Inhibition was quantified by calculating the width of the E. coli growth inhibition ring as (*D* − *d*)/2, where *D* is the diameter of the E. coli growth inhibition ring, and *d* is the diameter of the X. albilineans culture, as described in previous work (12). We measured 15 inhibition halos per strain.

### DNA preparation and genome sequencing.

**(i) DNA preparation.** Whole-genome sequencing of the isolates was performed with the MinION platform (38). The libraries were prepared using the SQK-LSK109 barcoding genomic DNA kit and sequenced, two by two, in R9.4.1 FLO-MIN106 flow cells, generating single-end reads. Strains Xa04 (barcode NB01) and Xa21 (barcode NB02), as well as strains Xa11 (barcode NB03) and Xa26 (barcode NB04), were sequenced together in 48-h runs. The reads underwent quality control and were separated according to the barcode adapters through METRICHOR software (39).

**(ii) Transcriptome sequencing (RNA-seq).** Freshly grown cultures of X. albilineans Xa4, Xa11, Xa21, and Xa26 were harvested and immediately subjected to RNA extraction. We used the Qiagen RNeasy Protect minikit (catalog no. 74124) for RNA extraction using chemical disruption of cells (proteinase K at 20 mg/mL and lysozyme at 15 mg/mL), following the manufacturer's recommendations. After RNA extraction, total RNA was treated with Turbo DNase (Thermo Fisher Scientific; catalog no. AM2238) according to the manufacturer's instructions. rRNA was depleted using the selective depletion method (40), with probes custom synthesized for *Xanthomonas* rRNA sequences.

The cDNA libraries were constructed using TruSeq Stranded Total RNA Library Prep Plant (Illumina, Inc., California, USA) according to the manufacturer´s instructions. Equimolar amounts (170 nM) of cDNA fragments of the four X. albilineans isolates labeled with different indices (IDT for Illumina, TruSeq RNA UD indices) were pooled and poured into one lane of the HiSeq 1500 System (Illumina) platform in order to obtain at least 20 million PE reads (~5 million PE reads/isolate), i.e., approximately 5% of the lane capacity. Sequencing was performed by using the c and TruSeq SBS kit v3-HS (200 cycles) (Illumina) for cluster generation and PE read sequencing of approximately 100 bases of each end.

Reads were demultiplexed by using BaseSpace sequence hub (Illumina) and trimmed on CLC Genomics Workbench v.20.0.4 (Qiagen) by using default parameters for cropping the adapters and for quality control, namely, a score limit of 0.05 and a maximum number of ambiguities equal to 2. Additionally, the five terminal bases of the 5′ and 3′ ends were discarded. PE reads were mapped onto their respective genomes with cost settings for mismatch, insertion, and deletion of 2, 3, and 3, respectively, and at least 80% coverage and 90% identity. The expression value was calculated in transcripts per million (TPM) values by using the RNA-Seq Analysis tool on CLC Genomics Workbench.

RNA-seq reads were also used for polishing the contigs obtained from genome sequencing by using the default parameters of the CLC tool Long Read Support/Polish with Reads, including window size of partial order alignments equal to 500 (41).

### Data analysis.

**(i) Genome assembly and polishing.** The *de novo* assembly of the genomes was performed using two methodologies. The genomes of isolates Xa04 and Xa11 were assembled with the Long Read Support module of the CLC Genomics Workbench v.20.0.2 software, which is a pipeline that uses the minimap2, miniasm, and Racon software in the assembly of long reads (alignment, assembly, and correction). The genomes of isolates Xa21 and Xa26 were assembled with the Canu v.2.0 software, a modular approach composed of three processing stages, correction, trimming, and assembly, performed in series (42). Then, the sequences were corrected with Illumina data obtained by RNA-seq of the strains grown *in vitro*. The correction was also realized using the Long Read Support module of the CLC Genomics Workbench v.20.0.2 software.

**(ii) Genome annotation.** Genome annotation was performed with the Prokka v.1.12 software, a pipeline that uses hierarchical methodology, curated databases, and several prediction tools to identify and assign a function to coding sequences (CDSs) and other genome structures, such as tRNA, rRNA, noncoding RNA (ncRNA), and CRISPR regions. Among the tools applied in the analysis are Prodigal, RNAmmer, Aragorn, SignalP, Infernal, and BLAST+ (43).

The insertion sequences (ISs) were predicted with ISsaga v.1.0; in our studies, we considered only the complete ISs (17). The prophage sequences were analyzed with PHASTER (18, 19). We performed a second prediction of transfer RNAs and CRISPR regions and their associated proteins using tRNAscan-SE v.2.0 (44) and CRISPRCasFinder v.1.1.2 (16), respectively.

**(iii) Transcriptomic analyses.** After mapping the reads and calculating the expression values in TPM, the data obtained were applied in a differential expression analysis. The determination of differentially expressed genes was based on the variation in ribosomal gene expression values; the expression data (TPM) of 54 ribosomal genes, identified in all genomes, were plotted in box plots specific to each genome, allowing the definition of the standard value range to be considered in the analysis.

Based on these results, genes whose expression values fell in the range of the ribosomal protein set were considered highly expressed genes, genes whose expression values were above this range were considered very highly expressed genes, and genes whose expression values were below this range were considered basally expressed.

**(iv) Average nucleotide identity and phylogenetic analysis.** The public genomic sequences of 17 worldwide strains of X. albilineans, including the reference genomes (GPE PC73 and Xa-FJ1 strains), in addition to the sequence of Xanthomonas sacchari R1 (used as an outgroup), were retrieved from the NCBI database (see Table S1 in the supplemental material). The average nucleotide identity (ANI) of 18 public *Xanthomonas* strains and the 4 Brazilian strains were measured by pairwise genome comparisons based on BLAST+ using JSpeciesWS Online Service (http://jspecies.ribohost.com/jspeciesws/) (45).

Orthologous gene group identification was performed with OrthoMCL v.2.0 (default parameters) (21). Two different clustering iterations were performed: the first used amino acid sequences of the complete X. albilineans genomes to identify core and specific genes, while the second used amino acid sequences of all the X. albilineans genomes and the X. sacchari R1 genome to determine the gene set shared between them. Based on the second clustering iteration results, 91 core single-copy genes, conserved among all genomes, were selected (Table S2). Sequences were aligned using MAFFT (in Seaview) and the IQ-TREE server (http://iqtree.cibiv.univie.ac.at) for the construction of a maximum likelihood, 1,000 bootstraps (46). In addition, the resulting tree was compared using the T-REX web server (http://www.trex.uqam.ca) for the construction of neighbor joining and parsimony, 1,000 bootstraps (47), but excluding X. sacchari as an outgroup. Tree topology remained unchanged in all cases (data not shown).

### Comparative genomic analysis and pan- and core genomes.

Global alignment between the Brazilian strains and the reference strain genomes was carried out with the progressiveMAUVE tool of the MAUVE software v.2.4.0 (48, 49). The analysis enabled the identification of highly similar genomic regions (colinear blocks), in addition to structural variations such as inversions, deletions, and insertions, visually characterized in a linear graphical representation.

The PAN genome of X. albilineans was determined with the PGAP-X module (http://pgaweb.vlcc.cn/pgapx/analyse) of the Pan-Genome Analysis Web Server tool (http://pgaweb.vlcc.cn/) (50, 51). In this analysis, only complete genomes could be used, which included the reference and the four Brazilian strains of genomes.

### Data availability.

All data are under BioProject accession number PRJNA792816. GenBank accession numbers for chromosome data are SAMN24462201 (Xa04), SAMN24462202 (Xa11), SAMN24462203 (Xa21), and SAMN24462204 (Xa26). RNA-seq data are available at SRA accession number SRR19025915 Xa04, SRR19025914 (Xa11), SRR19025913 (Xa21), and SRR19025912 (Xa26).
