# Supplementary material for: Genome Organization of Four Brazilian Xanthomonas albilineans Strains Does Not Correlate with Aggressiveness
Source: Microbiol Spectr. 2023 Apr 13;11(3):e02802-22. doi: 10.1128/spectrum.02802-22 (PMC10269729; doi:10.1128/spectrum.02802-22)
Supplement: Supplemental file 1 — Supplemental material. Download spectrum.02802-22-s0001.pdf, PDF file, 0.2 MB [file spectrum.02802-22-s0001.pdf]

Supplementary Material:

Table S1: General information about public *Xanthomonas* strains used in this study.

Table S2: 91 core single copy genes selected for phylogenetic analyses.

Table S3: List of ribosomal proteins and common protein genes classified as highly expressed in the *X. albilineans* strains.

Organism	Strain	Size (Mb)	BioSample	%GC	Assembly	Scaffolds	CDS	Geographic Location	Collection	Reference
	Xa-FJ1	3.75612	SAMN12905433	62.97%	Complete	2	2968	China	2015	Zhang et al. 2020
	GPE PC73	3.85230	SAMEA3138291	62.91%	Complete	4	3105	Guadeloupe	2003	Pieretti et al., 2009
	HVO005	3.65265	SAMN03262630	62.90%	Scaffold	2	2912	Burkina Faso	1979	Royer et al. 2015
	HVO082	3.63602	SAMN03262511	63.00%	Scaffold	1	2878	Burkina Faso	1989	Royer et al. 2015
	CFBP2523	3.68418	SAMN05526486	63.10%	Scaffold	148	2996	Fiji	1961	Merda et al., 2016
	FIJ080	3.68125	SAMN03257616	63.00%	Scaffold	1	2929	Fiji	1961	Royer et al. 2015
	REU209	3.68727	SAMN03262627	63.00%	Scaffold	2	2921	France	1995	Royer et al. 2015
	REU174	3.86323	SAMN03263614	62.80%	Scaffold	29	3097	France	1995	Royer et al. 2015
Xanthomonas albilineans	GAB266	3.79190	SAMN03263609	62.90%	Scaffold	4	3013	Gabon	2011	Royer et al. 2015
	GPE PC86	3.85143	SAMN03262651	62.80%	Scaffold	4	3158	Guadeloupe	2003	Royer et al. 2015
	GPE PC17	3.81135	SAMN03262650	62.80%	Scaffold	4	3122	Guadeloupe	2003	Royer et al. 2015
	MTQ032	3.80155	SAMN03257639	62.90%	Scaffold	4	3079	Martinique	1932	Royer et al. 2015
	PNG130	3.54265	SAMN03262494	63.30%	Scaffold	4	2868	Papua New Guinea	1993	Royer et al. 2015
	LKA070	3.66795	SAMN03262508	63.10%	Scaffold	2	2940	Sri Lanka	1962	Royer et al. 2015
	USA048	3.58204	SAMN03262645	63.10%	Scaffold	6	2828	USA	1986	Royer et al. 2015
	Xa23R1	3.54903	SAMN03262642	63.10%	Scaffold	4	2839	USA	1993	Pieretti et al., 2015
	XaFL07-1	3.79895	SAMN03262647	62.90%	Scaffold	6	3111	USA	2007	Royer et al. 2015
Xanthomonas sacchari	R1	5.00611	SAMN03273272	68.96%	Complete	2	3944	China	2011	Fang et al., 2014

Fang,Y., Lin,H., Wu,L., Ren,D., Ye,W., Dong,G., Zhu,L. and Guo,L. 2014. Genome sequence of Xanthomonas sacchari R1, a bacterium isolated from the rice seed. **Unpublished**. State Key Lab for Rice Biology, China National Rice Research Institute, Zhejiang University, Hangzhou, China, Hangzhou, Hangzhou 310006, China.
Merda,D., Briand,M., Bosis,E., Rousseau,C., Portier,P., Jacques,M.-A. and Fischer-Le Saux,M. 2016. Evolution of the type three secretion system and type three effector repertoires in Xanthomonas. **Unpublished**. SPE, INRA, rue Georges Morel, Beaucouze 49071, France.
Pieretti,I., Cociancich,S., Bolot,S., Carrere,S., Morisset,A., Rott,P. and Royer,M. 2015. Full Genome Sequence Analysis of Two Isolates Reveals a Novel Xanthomonas Species Close to the Sugarcane Pathogen Xanthomonas albilineans. **Genes** (Basel) 6(3):714-733.
Pieretti,I., Royer,M., Barbe,V., Carrere,S., Koebnik,R., Cociancich,S., Couloux,A., Darrasse,A., Gouzy,J., Jacques,M.A., Lauber,E., Manceau,C., Mangenot,S., Poussier,S., Segurens,B., Szurek,B., Verdier,V., Arlat,M. and Rott,P. 2009. The complete genome sequence of Xanthomonas albilineans provides new insights into the reductive genome evolution of the xylem-limited Xanthomonadaceae. **BMC Genomics** 10:616.
Royer,M., Cociancich,S., Rott,P., Barbe,V., Bolot,S., Carrere,S. and Pieretti,I. 2015. **NCBI Direct Submission**. Laboratoire des Interactions Plantes Micro-organismes, INRA/CNRS, Chemin de Borderouge, Castanet-Tolosan 31200, France.
Zhang,H.-L., Ntambo,M.S., Rott,P.C., Chen,G., Chen,L.-L., Huang,M.-T. and Gao,S.-J. 2020. Complete Genome Sequence Reveals Evolutionary and Comparative Genomic Features of Xanthomonas albilineans Causing Sugarcane Leaf Scald. **Microorganisms** 8(2):182.

			ID_RefSeq or Locus_Tag																					
			Xanthomonas albilineans																					*Xanthomonas sacchari*
**Ortholog_Group**	**GeneID**	**Product**	**PNG130**	**FIJ080**	**Xa23R1**	**USA048**	**REU174**	**LKA070**	**Xa-FJ1**	**Xa04**	**Xa11**	**Xa21**	**Xa26**	**GPEPC73**	**GPEPC17**	**GPEPC86**	**MTQ032**	**XaFL07-1**	**HVO005**	**GAB266**	**HVO082**	**REU209**	**CFBP2523**	**R1**
OrtGroup123	Gene01	50S ribosomal protein L28	WP_010342733.1	WP_010342733.1	WP_010342733.1	WP_010342733.1	WP_010342733.1	WP_010342733.1	WP_010342733.1	Xa04_00580	Xa11_00575	Xa21_00575	Xa26_00575	WP_010342733.1	WP_010342733.1	WP_010342733.1	WP_010342733.1	WP_010342733.1	WP_010342733.1	WP_010342733.1	WP_010342733.1	WP_010342733.1	WP_010342733.1	WP_010342733.1
OrtGroup136	Gene02	biopolymer transporter ExbD	WP_012914575.1	WP_012914575.1	WP_012914575.1	WP_012914575.1	WP_012914575.1	WP_012914575.1	WP_012914575.1	Xa04_00050	Xa11_00050	Xa21_00050	Xa26_00050	WP_012914575.1	WP_012914575.1	WP_012914575.1	WP_012914575.1	WP_012914575.1	WP_012914575.1	WP_012914575.1	WP_012914575.1	WP_012914575.1	WP_012914575.1	WP_043093888.1
OrtGroup162	Gene03	hypothetical protein	WP_045769443.1	WP_045765033.1	WP_045766935.1	WP_045766935.1	WP_045757538.1	WP_045761619.1	WP_012914608.1	Xa04_00225	Xa11_00225	Xa21_00225	Xa26_00225	WP_012914608.1	WP_012914608.1	WP_012914608.1	WP_012914608.1	WP_012914608.1	WP_045761619.1	WP_045760302.1	WP_045760302.1	WP_045760302.1	WP_045765033.1	WP_052250814.1
OrtGroup179	Gene05	hypothetical protein	WP_012914635.1	WP_045765047.1	WP_045766950.1	WP_045766950.1	WP_012914635.1	WP_012914635.1	WP_012914635.1	Xa04_00375	Xa11_00370	Xa21_00370	Xa26_00370	WP_012914635.1	WP_012914635.1	WP_012914635.1	WP_012914635.1	WP_012914635.1	WP_012914635.1	WP_012914635.1	WP_012914635.1	WP_012914635.1	WP_045765047.1	WP_043093762.1
OrtGroup2702	Gene06	hypothetical protein	WP_052689753.1	WP_155394263.1	WP_152624993.1	WP_152624993.1	WP_152624993.1	WP_152624993.1	WP_155394263.1	Xa04_00525	Xa11_00520	Xa21_00520	Xa26_00520	WP_012914663.1	WP_012914663.1	WP_012914663.1	WP_155394263.1	WP_012914663.1	WP_152624993.1	WP_155394263.1	WP_155394263.1	WP_155393038.1	WP_012914663.1	WP_156117257.1
OrtGroup2563	Gene08	YifB family Mg chelatase-like AAA ATPase	WP_045769609.1	WP_045765186.1	WP_045765186.1	WP_045765186.1	WP_045757616.1	WP_045760387.1	WP_045757616.1	Xa04_01500	Xa11_02045	Xa21_02145	Xa26_01850	WP_012914917.1	WP_012914917.1	WP_012914917.1	WP_012914917.1	WP_012914917.1	WP_045760387.1	WP_045760387.1	WP_045760387.1	WP_045771447.1	WP_045765186.1	WP_043094330.1
OrtGroup364	Gene09	SURF1 family protein	WP_045769624.1	WP_012914959.1	WP_045767054.1	WP_045767054.1	WP_012914959.1	WP_012914959.1	WP_012914959.1	Xa04_01740	Xa11_02280	Xa21_02380	Xa26_02085	WP_012914959.1	WP_012914959.1	WP_012914959.1	WP_012914959.1	WP_012914959.1	WP_012914959.1	WP_012914959.1	WP_012914959.1	WP_012914959.1	WP_012914959.1	WP_052250868.1
OrtGroup370	Gene10	beta-glucosidase	WP_045769630.1	WP_045765215.1	WP_045765215.1	WP_045765215.1	WP_012914971.1	WP_012914971.1	WP_012914971.1	Xa04_01805	Xa11_02345	Xa21_02445	Xa26_02150	WP_012914971.1	WP_012914971.1	WP_012914971.1	WP_012914971.1	WP_012914971.1	WP_012914971.1	WP_012914971.1	WP_012914971.1	WP_012914971.1	WP_045765215.1	WP_043094378.1
OrtGroup383	Gene11	glycerol-3-phosphate dehydrogenase	WP_045769639.1	WP_045765231.1	WP_045767064.1	WP_045767064.1	WP_045758716.1	WP_045758716.1	WP_012914992.1	Xa04_01910	Xa11_02450	Xa21_02550	Xa26_02255	WP_012914992.1	WP_012914992.1	WP_012914992.1	WP_012914992.1	WP_012914992.1	WP_045758716.1	WP_045758716.1	WP_045758716.1	WP_045758716.1	WP_045765231.1	WP_043094440.1
OrtGroup388	Gene12	fumarylacetoacetate hydrolase family protein	WP_045765235.1	WP_045765235.1	WP_045765235.1	WP_045765235.1	WP_045757638.1	WP_045757638.1	WP_012914998.1	Xa04_01940	Xa11_02480	Xa21_02580	Xa26_02285	WP_012914998.1	WP_012914998.1	WP_012914998.1	WP_012914998.1	WP_012914998.1	WP_045757638.1	WP_045757638.1	WP_045757638.1	WP_045757638.1	WP_045765235.1	WP_043094450.1
OrtGroup437	Gene13	50S ribosomal protein L10	WP_012915051.1	WP_012915051.1	WP_012915051.1	WP_012915051.1	WP_045757659.1	WP_012915051.1	WP_012915051.1	Xa04_02230	Xa11_02770	Xa21_02870	Xa26_02575	WP_012915051.1	WP_012915051.1	WP_012915051.1	WP_012915051.1	WP_012915051.1	WP_012915051.1	WP_012915051.1	WP_012915051.1	WP_012915051.1	WP_012915051.1	WP_010341340.1
OrtGroup439	Gene14	DNA-directed RNA polymerase subunit beta	WP_045769665.1	WP_012915053.1	WP_045771343.1	WP_045767081.1	WP_012915053.1	WP_012915053.1	WP_012915053.1	Xa04_02240	Xa11_02780	Xa21_02880	Xa26_02585	WP_012915053.1	WP_012915053.1	WP_012915053.1	WP_012915053.1	WP_012915053.1	WP_012915053.1	WP_012915053.1	WP_012915053.1	WP_012915053.1	WP_012915053.1	WP_017908393.1
OrtGroup462	Gene15	preprotein translocase subunit SecY	WP_045757663.1	WP_045757663.1	WP_045767084.1	WP_045767084.1	WP_045757663.1	WP_045757663.1	WP_012915076.1	Xa04_02375	Xa11_02915	Xa21_03015	Xa26_02720	WP_012915076.1	WP_012915076.1	WP_012915076.1	WP_012915076.1	WP_012915076.1	WP_045757663.1	WP_045757663.1	WP_045757663.1	WP_045757663.1	WP_045757663.1	WP_043093027.1
OrtGroup464	Gene16	DNA-directed RNA polymerase subunit alpha	WP_012915078.1	WP_012915078.1	WP_012915078.1	WP_012915078.1	WP_045757664.1	WP_045757664.1	WP_012915078.1	Xa04_02400	Xa11_02935	Xa21_03035	Xa26_02740	WP_012915078.1	WP_012915078.1	WP_012915078.1	WP_012915078.1	WP_012915078.1	WP_045757664.1	WP_045757664.1	WP_045757664.1	WP_045757664.1	WP_012915078.1	WP_010340455.1
OrtGroup475	Gene17	glycerophosphodiester phosphodiesterase family protein	WP_045769680.1	WP_045765270.1	WP_045765270.1	WP_045765270.1	WP_045757672.1	WP_012915091.1	WP_012915091.1	Xa04_02470	Xa11_03005	Xa21_03105	Xa26_02810	WP_012915091.1	WP_012915091.1	WP_012915091.1	WP_012915091.1	WP_012915091.1	WP_045761706.1	WP_012915091.1	WP_012915091.1	WP_012915091.1	WP_045765270.1	WP_043093006.1
OrtGroup480	Gene18	OmpA family protein	WP_045760419.1	WP_045760419.1	WP_045767091.1	WP_045767091.1	WP_012915097.1	WP_012915097.1	WP_012915097.1	Xa04_02500	Xa11_03035	Xa21_03140	Xa26_02840	WP_012915097.1	WP_012915097.1	WP_012915097.1	WP_012915097.1	WP_012915097.1	WP_045760419.1	WP_045760419.1	WP_045760419.1	WP_045760419.1	WP_045760419.1	WP_043092989.1
OrtGroup500	Gene19	isocitrate dehydrogenase	WP_045769697.1	WP_045764259.1	WP_045764259.1	WP_045764259.1	WP_012915119.1	WP_045764259.1	WP_012915119.1	Xa04_02615	Xa11_03150	Xa21_03260	Xa26_02955	WP_012915119.1	WP_012915119.1	WP_012915119.1	WP_012915119.1	WP_012915119.1	WP_012915119.1	WP_012915119.1	WP_012915119.1	WP_012915119.1	WP_045764259.1	WP_043092958.1
OrtGroup517	Gene20	molybdenum cofactor biosynthesis protein MoaE	WP_045769704.1	WP_045765293.1	WP_045765293.1	WP_045765293.1	WP_045757689.1	WP_045760431.1	WP_012915140.1	Xa04_02770	Xa11_03305	Xa21_03415	Xa26_03110	WP_012915140.1	WP_012915140.1	WP_012915140.1	WP_012915140.1	WP_012915140.1	WP_045760431.1	WP_045760431.1	WP_045760431.1	WP_045760431.1	WP_045765293.1	WP_043092944.1
OrtGroup554	Gene21	phospholipase C, phosphocholine-specific	WP_080932956.1	WP_080928041.1	WP_080934242.1	WP_080934242.1	WP_012915186.1	WP_080929668.1	WP_012915186.1	Xa04_03010	Xa11_03550	Xa21_03655	Xa26_03350	WP_012915186.1	WP_012915186.1	WP_012915186.1	WP_012915186.1	WP_012915186.1	WP_080932397.1	WP_080932397.1	WP_080932397.1	WP_080932397.1	WP_080928041.1	WP_156117324.1
OrtGroup591	Gene22	sterol desaturase family protein	WP_045769760.1	WP_045765349.1	WP_045767162.1	WP_045767162.1	WP_052687842.1	WP_012915245.1	WP_012915245.1	Xa04_03330	Xa11_03870	Xa21_03965	Xa26_03670	WP_012915245.1	WP_012915245.1	WP_012915245.1	WP_012915245.1	WP_012915245.1	WP_052687842.1	WP_052687842.1	WP_052687842.1	WP_052687842.1	WP_045765349.1	WP_043091775.1
OrtGroup2258	Gene23	response regulator	WP_045769772.1	WP_045765361.1	WP_045767170.1	WP_045767170.1	WP_045757792.1	WP_045760465.1	WP_045756730.1	Xa04_03430	Xa11_03975	Xa21_04065	Xa26_03785	WP_012915264.1	WP_012915264.1	WP_045756730.1	WP_045756730.1	WP_045756730.1	WP_045760465.1	WP_045760465.1	WP_045760465.1	WP_045760465.1	WP_045765361.1	WP_043094797.1
OrtGroup631	Gene24	hypothetical protein	WP_012915294.1	WP_012915294.1	WP_012915294.1	WP_012915294.1	WP_045757803.1	WP_012915294.1	WP_012915294.1	Xa04_03585	Xa11_04130	Xa21_04220	Xa26_03940	WP_012915294.1	WP_012915294.1	WP_012915294.1	WP_012915294.1	WP_012915294.1	WP_012915294.1	WP_012915294.1	WP_012915294.1	WP_012915294.1	WP_012915294.1	WP_043094848.1
OrtGroup632	Gene25	6-phosphofructokinase	WP_012915295.1	WP_012915295.1	WP_012915295.1	WP_012915295.1	WP_045757804.1	WP_012915295.1	WP_012915295.1	Xa04_03590	Xa11_04135	Xa21_04225	Xa26_03945	WP_012915295.1	WP_012915295.1	WP_012915295.1	WP_012915295.1	WP_012915295.1	WP_012915295.1	WP_012915295.1	WP_012915295.1	WP_012915295.1	WP_012915295.1	WP_043094850.1
OrtGroup685	Gene26	pteridine reductase	WP_045769812.1	WP_012915361.1	WP_012915361.1	WP_012915361.1	WP_012915361.1	WP_045761742.1	WP_012915361.1	Xa04_03925	Xa11_04480	Xa21_04560	Xa26_04285	WP_012915361.1	WP_012915361.1	WP_012915361.1	WP_012915361.1	WP_012915361.1	WP_045761742.1	WP_045760492.1	WP_045760492.1	WP_045760492.1	WP_012915361.1	WP_043094745.1
OrtGroup730	Gene27	polyketide cyclase	WP_045769847.1	WP_012915427.1	WP_045767239.1	WP_045767239.1	WP_012915427.1	WP_045764327.1	WP_012915427.1	Xa04_04265	Xa11_04815	Xa21_04895	Xa26_04620	WP_012915427.1	WP_012915427.1	WP_012915427.1	WP_012915427.1	WP_012915427.1	WP_012915427.1	WP_012915427.1	WP_012915427.1	WP_012915427.1	WP_012915427.1	WP_043092831.1
OrtGroup735	Gene28	formate-dependent phosphoribosylglycinamide formyltransferase	WP_045769852.1	WP_045765444.1	WP_045767244.1	WP_045767244.1	WP_045757858.1	WP_045757858.1	WP_012915434.1	Xa04_04305	Xa11_04850	Xa21_04930	Xa26_04655	WP_012915434.1	WP_012915434.1	WP_012915434.1	WP_012915434.1	WP_012915434.1	WP_045760512.1	WP_045760512.1	WP_045760512.1	WP_045760512.1	WP_045765444.1	WP_043092815.1
OrtGroup740	Gene29	enoyl-CoA hydratase/isomerase family protein	WP_045767248.1	WP_045765447.1	WP_045767248.1	WP_045767248.1	WP_012915440.1	WP_012915440.1	WP_012915440.1	Xa04_04335	Xa11_04880	Xa21_04960	Xa26_04685	WP_012915440.1	WP_012915440.1	WP_012915440.1	WP_012915440.1	WP_012915440.1	WP_012915440.1	WP_012915440.1	WP_012915440.1	WP_012915440.1	WP_045765447.1	WP_043092802.1
OrtGroup747	Gene30	murein biosynthesis integral membrane protein MurJ	WP_104629934.1	WP_104629934.1	WP_152625141.1	WP_152625141.1	WP_155394884.1	WP_155393974.1	WP_012915448.1	Xa04_04375	Xa11_04920	Xa21_05000	Xa26_04725	WP_012915448.1	WP_155393731.1	WP_155393731.1	WP_155393731.1	WP_155393731.1	WP_155393179.1	WP_155393179.1	WP_155393179.1	WP_155393179.1	WP_104629934.1	WP_043092794.1
OrtGroup760	Gene31	ABC transporter permease	WP_045757870.1	WP_045765467.1	WP_045767260.1	WP_045767260.1	WP_045757870.1	WP_045760520.1	WP_012915470.1	Xa04_04485	Xa11_05030	Xa21_05110	Xa26_04835	WP_012915470.1	WP_012915470.1	WP_012915470.1	WP_012915470.1	WP_012915470.1	WP_045760520.1	WP_045760520.1	WP_045760520.1	WP_045760520.1	WP_045765467.1	WP_043092768.1
OrtGroup775	Gene32	dCTP deaminase	WP_012915496.1	WP_012915496.1	WP_012915496.1	WP_012915496.1	WP_012915496.1	WP_012915496.1	WP_012915496.1	Xa04_04610	Xa11_05150	Xa21_05230	Xa26_04955	WP_012915496.1	WP_012915496.1	WP_012915496.1	WP_012915496.1	WP_012915496.1	WP_012915496.1	WP_012915496.1	WP_012915496.1	WP_012915496.1	WP_012915496.1	WP_010340509.1
OrtGroup778	Gene33	transcription elongation factor GreB	WP_045769889.1	WP_045765481.1	WP_045767274.1	WP_045767274.1	WP_012915501.1	WP_012915501.1	WP_012915501.1	Xa04_04635	Xa11_05175	Xa21_05255	Xa26_04980	WP_012915501.1	WP_012915501.1	WP_012915501.1	WP_012915501.1	WP_012915501.1	WP_012915501.1	WP_012915501.1	WP_012915501.1	WP_012915501.1	WP_045765481.1	WP_017915170.1
OrtGroup783	Gene34	D-glycerate dehydrogenase	WP_045770935.1	WP_045766663.1	WP_045768234.1	WP_045768234.1	WP_012915507.1	WP_012915507.1	WP_012915507.1	Xa04_04665	Xa11_05205	Xa21_05285	Xa26_05010	WP_012915507.1	WP_012915507.1	WP_012915507.1	WP_012915507.1	WP_012915507.1	WP_012915507.1	WP_045761227.1	WP_045761227.1	WP_045761227.1	WP_045766663.1	WP_043092582.1
OrtGroup810	Gene35	NADH-quinone oxidoreductase subunit M	WP_012915536.1	WP_012915536.1	WP_012915536.1	WP_012915536.1	WP_012915536.1	WP_045764351.1	WP_012915536.1	Xa04_04825	Xa11_05370	Xa21_05445	Xa26_05170	WP_012915536.1	WP_012915536.1	WP_012915536.1	WP_012915536.1	WP_012915536.1	WP_045760536.1	WP_045760536.1	WP_045760536.1	WP_045760536.1	WP_012915536.1	WP_043092566.1
OrtGroup872	Gene36	cold-shock protein	WP_012915623.1	WP_012915623.1	WP_045767332.1	WP_045767332.1	WP_012915623.1	WP_012915623.1	WP_012915623.1	Xa04_05365	Xa11_05890	Xa21_05950	Xa26_05670	WP_012915623.1	WP_012915623.1	WP_012915623.1	WP_012915623.1	WP_012915623.1	WP_012915623.1	WP_012915623.1	WP_012915623.1	WP_012915623.1	WP_012915623.1	WP_043092266.1
OrtGroup888	Gene37	ABC transporter ATP-binding protein	WP_045770949.1	WP_045765548.1	WP_045767346.1	WP_045767346.1	WP_012915646.1	WP_045764380.1	WP_012915646.1	Xa04_05490	Xa11_06010	Xa21_06070	Xa26_05790	WP_012915646.1	WP_012915646.1	WP_012915646.1	WP_012915646.1	WP_012915646.1	WP_045761784.1	WP_080933383.1	WP_080933383.1	WP_045761784.1	WP_045765548.1	WP_043092233.1
OrtGroup897	Gene38	replicative DNA helicase	WP_012915657.1	WP_012915657.1	WP_012915657.1	WP_012915657.1	WP_012915657.1	WP_012915657.1	WP_012915657.1	Xa04_05545	Xa11_06065	Xa21_06125	Xa26_05845	WP_012915657.1	WP_012915657.1	WP_012915657.1	WP_012915657.1	WP_012915657.1	WP_012915657.1	WP_012915657.1	WP_012915657.1	WP_012915657.1	WP_012915657.1	WP_017909018.1
OrtGroup927	Gene39	bifunctional hydroxymethylpyrimidine kinase/phosphomethylpyrimidine kinase	WP_045769982.1	WP_045766683.1	WP_045766683.1	WP_045766683.1	WP_045758784.1	WP_045764892.1	WP_012915695.1	Xa04_05740	Xa11_06260	Xa21_06325	Xa26_06040	WP_012915695.1	WP_012915695.1	WP_012915695.1	WP_012915695.1	WP_012915695.1	WP_045761250.1	WP_045761250.1	WP_045761250.1	WP_045761250.1	WP_045766683.1	WP_043091875.1
OrtGroup933	Gene40	ferredoxin family protein	WP_012915703.1	WP_012915703.1	WP_012915703.1	WP_012915703.1	WP_012915703.1	WP_012915703.1	WP_012915703.1	Xa04_05785	Xa11_06300	Xa21_06365	Xa26_06080	WP_012915703.1	WP_012915703.1	WP_012915703.1	WP_012915703.1	WP_012915703.1	WP_012915703.1	WP_012915703.1	WP_012915703.1	WP_012915703.1	WP_012915703.1	WP_017909525.1
OrtGroup2722	Gene41	9-O-acetylesterase	WP_045769987.1	WP_045765577.1	WP_045767376.1	WP_045767376.1	WP_012915710.1	WP_045761792.1	WP_161525213.1	Xa04_05820	Xa11_06335	Xa21_06400	Xa26_06115	WP_012915710.1	WP_012915710.1	WP_012915710.1	WP_012915710.1	WP_012915710.1	WP_045761792.1	WP_045760613.1	WP_045760613.1	WP_045761792.1	WP_045765577.1	WP_043091883.1
OrtGroup1037	Gene43	chemotaxis response regulator protein-glutamate methylesterase	WP_012915865.1	WP_012915865.1	WP_045767427.1	WP_045767427.1	WP_012915865.1	WP_012915865.1	WP_012915865.1	Xa04_06610	Xa11_07115	Xa21_07185	Xa26_06900	WP_012915865.1	WP_012915865.1	WP_012915865.1	WP_012915865.1	WP_012915865.1	WP_012915865.1	WP_012915865.1	WP_012915865.1	WP_012915865.1	WP_012915865.1	WP_026143996.1
OrtGroup1059	Gene44	flagellar biosynthetic protein FliR	WP_012915895.1	WP_012915895.1	WP_012915895.1	WP_012915895.1	WP_012915895.1	WP_012915895.1	WP_012915895.1	Xa04_06750	Xa11_07250	Xa21_07325	Xa26_07045	WP_012915895.1	WP_012915895.1	WP_012915895.1	WP_012915895.1	WP_012915895.1	WP_012915895.1	WP_012915895.1	WP_012915895.1	WP_012915895.1	WP_012915895.1	WP_017909008.1
OrtGroup1097	Gene45	flagellar basal body rod protein FlgC	WP_012915938.1	WP_012915938.1	WP_012915938.1	WP_012915938.1	WP_012915938.1	WP_012915938.1	WP_012915938.1	Xa04_06970	Xa11_07470	Xa21_07540	Xa26_07260	WP_012915938.1	WP_012915938.1	WP_012915938.1	WP_012915938.1	WP_012915938.1	WP_012915938.1	WP_012915938.1	WP_012915938.1	WP_012915938.1	WP_012915938.1	WP_017908570.1
OrtGroup1117	Gene46	voltage-gated chloride channel family protein	WP_045770080.1	WP_045765706.1	WP_045767453.1	WP_045767453.1	WP_012915965.1	WP_012915965.1	WP_012915965.1	Xa04_07110	Xa11_07610	Xa21_07680	Xa26_07400	WP_012915965.1	WP_012915965.1	WP_012915965.1	WP_012915965.1	WP_012915965.1	WP_045761823.1	WP_012915965.1	WP_012915965.1	WP_012915965.1	WP_045765706.1	WP_052250731.1
OrtGroup1152	Gene47	efflux RND transporter periplasmic adaptor subunit	WP_045770106.1	WP_045765764.1	WP_045767513.1	WP_045767513.1	WP_045758036.1	WP_045764462.1	WP_012916063.1	Xa04_07615	Xa11_08110	Xa21_08385	Xa26_07845	WP_012916063.1	WP_012916063.1	WP_012916063.1	WP_012916063.1	WP_012916063.1	WP_012916063.1	WP_012916063.1	WP_012916063.1	WP_012916063.1	WP_045765764.1	WP_043092063.1
OrtGroup1165	Gene48	glutamine-hydrolyzing GMP synthase	WP_012916081.1	WP_045765766.1	WP_045767522.1	WP_045767522.1	WP_012916081.1	WP_045764484.1	WP_012916081.1	Xa04_07760	Xa11_08245	Xa21_08520	Xa26_07985	WP_012916081.1	WP_012916081.1	WP_012916081.1	WP_012916081.1	WP_012916081.1	WP_045760706.1	WP_045760706.1	WP_045760706.1	WP_045760706.1	WP_045765766.1	WP_010342916.1
OrtGroup1178	Gene49	3-hydroxyacyl-CoA dehydrogenase/enoyl-CoA hydratase family protein	WP_045770115.1	WP_045765771.1	WP_045767528.1	WP_045767528.1	WP_045758044.1	WP_012916096.1	WP_012916096.1	Xa04_07835	Xa11_08320	Xa21_08595	Xa26_08060	WP_012916096.1	WP_012916096.1	WP_012916096.1	WP_012916096.1	WP_012916096.1	WP_045761845.1	WP_012916096.1	WP_012916096.1	WP_012916096.1	WP_045765771.1	WP_043092102.1
OrtGroup1226	Gene50	hypothetical protein	WP_045764495.1	WP_045765816.1	WP_045765816.1	WP_045765816.1	WP_012916161.1	WP_045764495.1	WP_012916161.1	Xa04_08180	Xa11_08665	Xa21_08940	Xa26_08410	WP_012916161.1	WP_012916161.1	WP_012916161.1	WP_012916161.1	WP_012916161.1	WP_012916161.1	WP_012916161.1	WP_012916161.1	WP_012916161.1	WP_045765816.1	WP_017915026.1
OrtGroup1266	Gene51	imidazolonepropionase	WP_045770170.1	WP_045765842.1	WP_045767574.1	WP_045767574.1	WP_045758100.1	WP_045758100.1	WP_012916213.1	Xa04_08465	Xa11_08945	Xa21_09220	Xa26_09030	WP_012916213.1	WP_012916213.1	WP_012916213.1	WP_012916213.1	WP_012916213.1	WP_045758100.1	WP_045758100.1	WP_045758100.1	WP_045758100.1	WP_045765842.1	WP_043095108.1
OrtGroup1315	Gene52	aminotransferase class III-fold pyridoxal phosphate-dependent enzyme	WP_080928109.1	WP_080928109.1	WP_080928109.1	WP_080928109.1	WP_012916278.1	WP_012916278.1	WP_012916278.1	Xa04_08795	Xa11_09275	Xa21_09550	Xa26_09360	WP_012916278.1	WP_012916278.1	WP_012916278.1	WP_012916278.1	WP_012916278.1	WP_012916278.1	WP_012916278.1	WP_012916278.1	WP_012916278.1	WP_080928109.1	WP_017908295.1
OrtGroup1356	Gene53	prepilin-type N-terminal cleavage/methylation domain-containing protein	WP_045770231.1	WP_012916361.1	WP_012916361.1	WP_012916361.1	WP_012916361.1	WP_012916361.1	WP_012916361.1	Xa04_09200	Xa11_09685	Xa21_09955	Xa26_09775	WP_012916361.1	WP_012916361.1	WP_012916361.1	WP_012916361.1	WP_012916361.1	WP_012916361.1	WP_012916361.1	WP_012916361.1	WP_012916361.1	WP_012916361.1	WP_043092345.1
OrtGroup1361	Gene54	glutamate 5-kinase	WP_045770234.1	WP_045765907.1	WP_045767624.1	WP_045767624.1	WP_012916375.1	WP_012916375.1	WP_012916375.1	Xa04_09290	Xa11_09775	Xa21_10045	Xa26_09865	WP_012916375.1	WP_012916375.1	WP_012916375.1	WP_012916375.1	WP_012916375.1	WP_012916375.1	WP_012916375.1	WP_012916375.1	WP_012916375.1	WP_045765907.1	WP_043095620.1
OrtGroup1406	Gene55	hypothetical protein	WP_045770260.1	WP_045765938.1	WP_045765938.1	WP_045765938.1	WP_045758194.1	WP_045764533.1	WP_012916440.1	Xa04_09625	Xa11_10115	Xa21_10385	Xa26_10200	WP_012916440.1	WP_012916440.1	WP_012916440.1	WP_012916440.1	WP_012916440.1	WP_012916440.1	WP_012916440.1	WP_012916440.1	WP_012916440.1	WP_045765938.1	WP_043092536.1
OrtGroup1407	Gene56	hypothetical protein	WP_012916441.1	WP_012916441.1	WP_012916441.1	WP_012916441.1	WP_012916441.1	WP_012916441.1	WP_012916441.1	Xa04_09630	Xa11_10120	Xa21_10390	Xa26_10205	WP_012916441.1	WP_012916441.1	WP_012916441.1	WP_012916441.1	WP_012916441.1	WP_012916441.1	WP_012916441.1	WP_012916441.1	WP_012916441.1	WP_012916441.1	WP_043092537.1
OrtGroup1422	Gene57	bifunctional 23S rRNA (guanine(2069)-N(7))-methyltransferase RlmK/23S rRNA (guanine(2445)-N(2))-methyltransferase RlmL	WP_045771006.1	WP_045766755.1	WP_045768319.1	WP_045768319.1	WP_045758204.1	WP_045762164.1	WP_012916462.1	Xa04_09740	Xa11_10230	Xa21_10500	Xa26_10315	WP_012916462.1	WP_012916462.1	WP_012916462.1	WP_012916462.1	WP_012916462.1	WP_045762164.1	WP_045761293.1	WP_045761293.1	WP_045761293.1	WP_045766755.1	WP_043092414.1
OrtGroup1446	Gene58	HlyD family efflux transporter periplasmic adaptor subunit	WP_045770283.1	WP_045760832.1	WP_045767669.1	WP_045767669.1	WP_045758215.1	WP_045760832.1	WP_012916492.1	Xa04_09900	Xa11_10390	Xa21_10660	Xa26_10475	WP_012916492.1	WP_012916492.1	WP_012916492.1	WP_012916492.1	WP_012916492.1	WP_045761899.1	WP_045760832.1	WP_045760832.1	WP_045760832.1	WP_045760832.1	WP_017913465.1
OrtGroup2686	Gene59	fimbrial biogenesis outer membrane usher protein	WP_155394168.1	WP_146084077.1	WP_152625075.1	WP_152625075.1	WP_155394826.1	WP_155393922.1	WP_155393678.1	Xa04_09975	Xa11_10465	Xa21_10735	Xa26_10550	WP_012916506.1	WP_155393678.1	WP_155393678.1	WP_155393678.1	WP_155393678.1	WP_155393554.1	WP_155393121.1	WP_155393121.1	WP_155393121.1	WP_146084077.1	WP_052250764.1
OrtGroup1512	Gene60	formate dehydrogenase accessory sulfurtransferase FdhD	WP_045770327.1	WP_045766013.1	WP_045767699.1	WP_045767699.1	WP_045758254.1	WP_012916587.1	WP_012916587.1	Xa04_10380	Xa11_10945	Xa21_11220	Xa26_10965	WP_012916587.1	WP_012916587.1	WP_012916587.1	WP_012916587.1	WP_012916587.1	WP_045760863.1	WP_045760863.1	WP_045760863.1	WP_045771499.1	WP_045766013.1	WP_043092402.1
OrtGroup1543	Gene61	arsenic transporter	WP_045767712.1	WP_045766033.1	WP_045767712.1	WP_045767712.1	WP_045758269.1	WP_045758269.1	WP_012916632.1	Xa04_10600	Xa11_11165	Xa21_11440	Xa26_11185	WP_012916632.1	WP_012916632.1	WP_012916632.1	WP_012916632.1	WP_012916632.1	WP_045758269.1	WP_045758269.1	WP_045758269.1	WP_045758269.1	WP_045766033.1	WP_043095736.1
OrtGroup1736	Gene62	hypothetical protein	WP_045770470.1	WP_045766184.1	WP_045766184.1	WP_045766184.1	WP_012916949.1	WP_045764657.1	WP_012916949.1	Xa04_12405	Xa11_13025	Xa21_13285	Xa26_12750	WP_012916949.1	WP_012916949.1	WP_012916949.1	WP_012916949.1	WP_012916949.1	WP_045760957.1	WP_045760957.1	WP_045760957.1	WP_045760957.1	WP_045766184.1	WP_082028754.1
OrtGroup2716	Gene63	autotransporter serine protease	WP_045770498.1	WP_045766209.1	WP_045767844.1	WP_045767844.1	WP_045758381.1	WP_045764667.1	WP_161525193.1	Xa04_12650	Xa11_13270	Xa21_13530	Xa26_12995	WP_012916998.1	WP_012916998.1	WP_012916998.1	WP_012916998.1	WP_012916998.1	WP_045761972.1	WP_045760972.1	WP_045760972.1	WP_045760972.1	WP_045766209.1	WP_052250772.1
OrtGroup2717	Gene64	cation transporter	WP_045770513.1	WP_045766225.1	WP_045767855.1	WP_045767855.1	WP_045758387.1	WP_155393946.1	WP_161525195.1	Xa04_12775	Xa11_13395	Xa21_13655	Xa26_13120	WP_012917023.1	WP_012917023.1	WP_045761499.1	WP_012917023.1	WP_012917023.1	WP_045761978.1	WP_045760983.1	WP_045760983.1	WP_045760983.1	WP_045766225.1	WP_043092738.1
OrtGroup1823	Gene65	PAS domain S-box protein	WP_045770532.1	WP_012917064.1	WP_012917064.1	WP_012917064.1	WP_012917064.1	WP_012917064.1	WP_012917064.1	Xa04_12980	Xa11_13600	Xa21_13855	Xa26_13320	WP_012917064.1	WP_012917064.1	WP_012917064.1	WP_012917064.1	WP_012917064.1	WP_012917064.1	WP_012917064.1	WP_012917064.1	WP_012917064.1	WP_012917064.1	WP_043094666.1
OrtGroup1825	Gene66	4-alpha-glucanotransferase	WP_045770535.1	WP_045766255.1	WP_045767873.1	WP_045767873.1	WP_012917067.1	WP_012917067.1	WP_012917067.1	Xa04_13000	Xa11_13620	Xa21_13875	Xa26_13340	WP_012917067.1	WP_012917067.1	WP_012917067.1	WP_012917067.1	WP_012917067.1	WP_012917067.1	WP_012917067.1	WP_012917067.1	WP_012917067.1	WP_045766255.1	WP_043094655.1
OrtGroup1826	Gene67	DUF2934 domain-containing protein	WP_012917069.1	WP_012917069.1	WP_012917069.1	WP_012917069.1	WP_012917069.1	WP_045760989.1	WP_012917069.1	Xa04_13020	Xa11_13635	Xa21_13885	Xa26_13350	WP_012917069.1	WP_012917069.1	WP_012917069.1	WP_012917069.1	WP_012917069.1	WP_045760989.1	WP_045760989.1	WP_045760989.1	WP_045760989.1	WP_012917069.1	WP_081613481.1
OrtGroup1831	Gene68	acyl-CoA desaturase	WP_012917074.1	WP_012917074.1	WP_045767877.1	WP_045767877.1	WP_045758085.1	WP_012917074.1	WP_012917074.1	Xa04_13045	Xa11_13665	Xa21_13915	Xa26_13380	WP_012917074.1	WP_012917074.1	WP_012917074.1	WP_012917074.1	WP_012917074.1	WP_012917074.1	WP_012917074.1	WP_012917074.1	WP_012917074.1	WP_012917074.1	WP_043094627.1
OrtGroup1832	Gene69	ferredoxin reductase	WP_045766259.1	WP_045766259.1	WP_045766259.1	WP_045766259.1	WP_012917075.1	WP_012917075.1	WP_012917075.1	Xa04_13050	Xa11_13670	Xa21_13920	Xa26_13385	WP_012917075.1	WP_012917075.1	WP_012917075.1	WP_012917075.1	WP_012917075.1	WP_012917075.1	WP_012917075.1	WP_012917075.1	WP_012917075.1	WP_045766259.1	WP_043094626.1
OrtGroup2718	Gene70	AGE family epimerase/isomerase	WP_045770544.1	WP_012917121.1	WP_045767881.1	WP_045767881.1	WP_012917121.1	WP_012917121.1	WP_161525197.1	Xa04_13310	Xa11_13925	Xa21_14180	Xa26_13630	WP_012917121.1	WP_012917121.1	WP_012917121.1	WP_012917121.1	WP_012917121.1	WP_012917121.1	WP_012917121.1	WP_012917121.1	WP_012917121.1	WP_012917121.1	WP_043094565.1
OrtGroup1871	Gene71	LPS export ABC transporter permease LptF	WP_045770556.1	WP_012917142.1	WP_045767898.1	WP_045767898.1	WP_045758406.1	WP_045758406.1	WP_012917142.1	Xa04_13420	Xa11_14035	Xa21_14290	Xa26_13740	WP_012917142.1	WP_012917142.1	WP_012917142.1	WP_012917142.1	WP_012917142.1	WP_045762003.1	WP_045758406.1	WP_045758406.1	WP_045758406.1	WP_012917142.1	WP_043093262.1
OrtGroup1881	Gene72	alpha-ketoglutarate-dependent dioxygenase AlkB	WP_045770564.1	WP_045766271.1	WP_045767906.1	WP_045767906.1	WP_045758411.1	WP_012917157.1	WP_012917157.1	Xa04_13495	Xa11_14115	Xa21_14365	Xa26_13815	WP_012917157.1	WP_012917157.1	WP_012917157.1	WP_012917157.1	WP_012917157.1	WP_045758411.1	WP_045758411.1	WP_045758411.1	WP_045758411.1	WP_045766271.1	WP_043093285.1
OrtGroup1927	Gene73	GGDEF domain-containing protein	WP_045770597.1	WP_045766304.1	WP_045767940.1	WP_045767940.1	WP_012917215.1	WP_045764744.1	WP_012917215.1	Xa04_13785	Xa11_14405	Xa21_14655	Xa26_14105	WP_012917215.1	WP_012917215.1	WP_012917215.1	WP_012917215.1	WP_012917215.1	WP_045761032.1	WP_045761032.1	WP_045761032.1	WP_045761032.1	WP_045766304.1	WP_043094479.1
OrtGroup1951	Gene74	TonB-dependent hemoglobin/transferrin/lactoferrin family receptor	WP_045770609.1	WP_045766319.1	WP_045767950.1	WP_045767950.1	WP_012917248.1	WP_045761038.1	WP_012917248.1	Xa04_13960	Xa11_14580	Xa21_14830	Xa26_14285	WP_012917248.1	WP_012917248.1	WP_012917248.1	WP_012917248.1	WP_012917248.1	WP_012917248.1	WP_045761038.1	WP_045761038.1	WP_045761038.1	WP_045766319.1	WP_043093127.1
OrtGroup2566	Gene75	hypothetical protein	WP_045766330.1	WP_045766330.1	WP_045766330.1	WP_045766330.1	WP_045758444.1	WP_045758444.1	WP_045758444.1	Xa04_14035	Xa11_14655	Xa21_14905	Xa26_14360	WP_012917263.1	WP_012917263.1	WP_012917263.1	WP_012917263.1	WP_012917263.1	WP_045762016.1	WP_045761043.1	WP_045761043.1	WP_045761043.1	WP_045766330.1	WP_043093155.1
OrtGroup2698	Gene76	undecaprenyldiphospho-muramoylpentapeptide beta-N-acetylglucosaminyltransferase	WP_155394251.1	WP_104629897.1	WP_152625163.1	WP_152625163.1	WP_155393203.1	WP_155393203.1	WP_155393748.1	Xa04_14130	Xa11_14750	Xa21_15000	Xa26_14455	WP_012917284.1	WP_155393748.1	WP_155393748.1	WP_155393748.1	WP_155393748.1	WP_155393203.1	WP_155394322.1	WP_155394322.1	WP_155393203.1	WP_104629897.1	WP_043093178.1
OrtGroup1989	Gene77	potassium-transporting ATPase subunit KdpC	WP_045770633.1	WP_045766350.1	WP_045767977.1	WP_045767977.1	WP_045758455.1	WP_045762021.1	WP_012917300.1	Xa04_14205	Xa11_14825	Xa21_15075	Xa26_14530	WP_012917300.1	WP_012917300.1	WP_012917300.1	WP_012917300.1	WP_012917300.1	WP_045762021.1	WP_012917300.1	WP_012917300.1	WP_012917300.1	WP_045766350.1	WP_043093193.1
OrtGroup2005	Gene78	MATE family efflux transporter	WP_012917341.1	WP_012917341.1	WP_012917341.1	WP_012917341.1	WP_012917341.1	WP_012917341.1	WP_012917341.1	Xa04_14410	Xa11_15030	Xa21_15280	Xa26_15030	WP_012917341.1	WP_012917341.1	WP_012917341.1	WP_012917341.1	WP_012917341.1	WP_045762025.1	WP_012917341.1	WP_012917341.1	WP_012917341.1	WP_012917341.1	WP_043094542.1
OrtGroup2046	Gene79	PAS domain S-box protein	WP_045770678.1	WP_045766405.1	WP_045768022.1	WP_045768022.1	WP_045758475.1	WP_045761078.1	WP_012917399.1	Xa04_14700	Xa11_15325	Xa21_15570	Xa26_15330	WP_012917399.1	WP_012917399.1	WP_012917399.1	WP_012917399.1	WP_045762320.1	WP_045761078.1	WP_045761078.1	WP_045761078.1	WP_045761078.1	WP_045766405.1	WP_017908767.1
OrtGroup2058	Gene80	dioxygenase	WP_045770691.1	WP_045766413.1	WP_045768034.1	WP_045768034.1	WP_012917424.1	WP_045764781.1	WP_012917424.1	Xa04_14820	Xa11_15435	Xa21_15690	Xa26_15440	WP_012917424.1	WP_012917424.1	WP_012917424.1	WP_012917424.1	WP_012917424.1	WP_045761085.1	WP_045761085.1	WP_045761085.1	WP_045761085.1	WP_045766413.1	WP_043093431.1
OrtGroup2182	Gene81	outer membrane protein assembly factor	WP_045771109.1	WP_045766898.1	WP_045766898.1	WP_045766898.1	WP_045758965.1	WP_012917607.1	WP_012917607.1	Xa04_15760	Xa11_16375	Xa21_16640	Xa26_16395	WP_012917607.1	WP_012917607.1	WP_012917607.1	WP_012917607.1	WP_012917607.1	WP_045761388.1	WP_045761388.1	WP_045761388.1	WP_045761388.1	WP_045766898.1	WP_043093721.1
OrtGroup2239	Gene82	bifunctional proline dehydrogenase/L-glutamate gamma-semialdehyde dehydrogenase PutA	WP_045769620.1	WP_045765205.1	WP_045767053.1	WP_045767053.1	WP_045757628.1	WP_045756695.1	WP_045756695.1	Xa04_01700	Xa11_02240	Xa21_02340	Xa26_02045	WP_045756695.1	WP_045756695.1	WP_045756695.1	WP_045756695.1	WP_045756695.1	WP_045761689.1	WP_045757628.1	WP_045757628.1	WP_045757628.1	WP_045765205.1	WP_043094364.1
OrtGroup2288	Gene83	bifunctional aspartate kinase/diaminopimelate decarboxylase	WP_045769915.1	WP_045765512.1	WP_045767290.1	WP_045767290.1	WP_045757885.1	WP_045757885.1	WP_045756766.1	Xa04_04950	Xa11_05495	Xa21_05570	Xa26_05295	WP_045756766.1	WP_045756766.1	WP_045756766.1	WP_045756766.1	WP_045756766.1	WP_045757885.1	WP_045757885.1	WP_045757885.1	WP_045757885.1	WP_045765512.1	WP_043095709.1
OrtGroup2340	Gene84	TrbI/VirB10 family protein	WP_045770225.1	WP_045756897.1	WP_045767613.1	WP_045767613.1	WP_045756897.1	WP_045756897.1	WP_045756897.1	Xa04_09075	Xa11_09560	Xa21_09825	Xa26_09645	WP_045756897.1	WP_045756897.1	WP_045756897.1	WP_045756897.1	WP_045756897.1	WP_045756897.1	WP_045756897.1	WP_045756897.1	WP_045756897.1	WP_045756897.1	WP_043092335.1
OrtGroup2440	Gene85	GTP-binding protein	WP_045770849.1	WP_045766587.1	WP_045768177.1	WP_045768177.1	WP_045757137.1	WP_045757137.1	WP_045757137.1	Xa04_00820	Xa11_00815	Xa21_00815	Xa26_00815	WP_045757137.1	WP_045757137.1	WP_045757137.1	WP_045757137.1	WP_045757137.1	WP_045757137.1	WP_045757137.1	WP_045757137.1	WP_045757137.1	WP_045766587.1	WP_043093673.1
OrtGroup2441	Gene86	bifunctional biotin--[acetyl-CoA-carboxylase] synthetase/biotin operon repressor	WP_155394089.1	WP_104629909.1	WP_152624998.1	WP_152624998.1	WP_155393042.1	WP_155393042.1	WP_045757152.1	Xa04_00945	Xa11_01265	Xa21_01265	Xa26_00935	WP_045757152.1	WP_012914813.1	WP_012914813.1	WP_012914813.1	WP_012914813.1	WP_155393042.1	WP_155393042.1	WP_155393042.1	WP_155393042.1	WP_104629909.1	WP_043095325.1
OrtGroup2442	Gene87	EamA family transporter RarD	WP_045757153.1	WP_045765117.1	WP_045757153.1	WP_045757153.1	WP_045757580.1	WP_045757153.1	WP_045757153.1	Xa04_00995	Xa11_01315	Xa21_01315	Xa26_01340	WP_045757153.1	WP_045757153.1	WP_045757153.1	WP_045757153.1	WP_045757153.1	WP_045757580.1	WP_045757580.1	WP_045757580.1	WP_045757580.1	WP_045765117.1	WP_043093605.1
OrtGroup2445	Gene88	TonB-dependent receptor	WP_045770886.1	WP_045766609.1	WP_045768186.1	WP_045768186.1	WP_045757162.1	WP_045764863.1	WP_045757162.1	Xa04_01550	Xa11_02090	Xa21_02190	Xa26_01895	WP_045757162.1	WP_045757162.1	WP_045757162.1	WP_045757162.1	WP_045757162.1	WP_045757162.1	WP_045757162.1	WP_045757162.1	WP_045757162.1	WP_045766609.1	WP_043094344.1
OrtGroup2458	Gene89	glutamate--cysteine ligase	WP_045770920.1	WP_045766644.1	WP_045768220.1	WP_045768220.1	WP_045757203.1	WP_045757203.1	WP_045757203.1	Xa04_04050	Xa11_04605	Xa21_04685	Xa26_04410	WP_045757203.1	WP_045757203.1	WP_045757203.1	WP_045757203.1	WP_045757203.1	WP_045761221.1	WP_045761221.1	WP_045761221.1	WP_045761221.1	WP_045766644.1	WP_043094767.1
OrtGroup2463	Gene90	methylamine utilization protein	WP_045766666.1	WP_045766666.1	WP_045768238.1	WP_045768238.1	WP_045758772.1	WP_045757216.1	WP_045757216.1	Xa04_04725	Xa11_05265	Xa21_05345	Xa26_05070	WP_045757216.1	WP_045757216.1	WP_045757216.1	WP_045757216.1	WP_045757216.1	WP_045761233.1	WP_045761233.1	WP_045761233.1	WP_045761233.1	WP_045766666.1	WP_043092574.1
OrtGroup2724	Gene91	PAS domain-containing protein	WP_045770979.1	WP_045766724.1	WP_045768289.1	WP_045768289.1	WP_045758799.1	WP_045764907.1	WP_161525217.1	Xa04_08040	Xa11_08525	Xa21_08800	Xa26_08270	WP_045758799.1	WP_045758799.1	WP_045758799.1	WP_045757279.1	WP_045762370.1	WP_045758799.1	WP_045758799.1	WP_045758799.1	WP_045758799.1	WP_045766724.1	WP_043092070.1
OrtGroup2587	Gene92	hypothetical protein	WP_155394110.1	WP_052687981.1	WP_152625015.1	WP_152625015.1	WP_155393064.1	WP_155393064.1	WP_049887205.1	Xa04_03885	Xa11_04440	Xa21_04520	Xa26_04245	WP_049887205.1	WP_049887205.1	WP_049887205.1	WP_049887205.1	WP_049887205.1	WP_155393524.1	WP_155393064.1	WP_155393064.1	WP_155393064.1	WP_052687981.1	WP_052250889.1
OrtGroup2614	Gene93	alpha/beta hydrolase	WP_080932934.1	WP_080928025.1	WP_080934236.1	WP_080934236.1	WP_080934102.1	WP_080929656.1	WP_080929656.1	Xa04_00265	Xa11_00265	Xa21_00265	Xa26_00265	WP_080929656.1	WP_080929656.1	WP_080929656.1	WP_080929656.1	WP_080929656.1	WP_080932383.1	WP_080932383.1	WP_080932383.1	WP_080932383.1	WP_080928025.1	WP_043095394.1
OrtGroup2617	Gene94	DUF3298 and DUF4163 domain-containing protein	WP_080932977.1	WP_080928066.1	WP_080929686.1	WP_080929686.1	WP_080929686.1	WP_080929686.1	WP_080929686.1	Xa04_05460	Xa11_05980	Xa21_06040	Xa26_05760	WP_080929686.1	WP_080929686.1	WP_080929686.1	WP_080929686.1	WP_080929686.1	WP_080929686.1	WP_080929686.1	WP_080929686.1	WP_080929686.1	WP_080928066.1	WP_043092245.1
